# Increasing the sensitivity and accuracy of detecting exosomes as biomarkers for cancer monitoring using optical nanobiosensors

**DOI:** 10.1186/s12935-024-03379-1

**Published:** 2024-05-30

**Authors:** Saman Yasamineh, Naghmeh Nikben, Mareb Hamed Ahmed, Radhwan Abdul Kareem, Ameer Kadhim Al-Aridhy, Mohammad Hosseini Hooshiar

**Affiliations:** 1 Young Researchers and Elite Club, Tabriz Branch, Islamic Azad University, Tabriz, Iran; 2 Iranian Cardiac Society, Tehran, Iran; 3 College of Dentistry, Al-Noor University, Mosul, Iraq; 4 Ahl Al Bayt University, Karbala, Iraq; 5 College of Health and Medical Technology, National University of Science and Technology, Dhi Qar, 64001 Iraq; 6 Department of Periodontics, School of Dentistry, Tehran University of Medical Sciences, Tehran, Iran

**Keywords:** Cancer, Exosomes, Optical biosensors, Nanoparticles, Detection

## Abstract

The advancement of nanoscience and material design in recent times has facilitated the creation of point-of-care devices for cancer diagnosis and biomolecule sensing. Exosomes (EXOs) facilitate the transfer of bioactive molecules between cancer cells and diverse cells in the local and distant microenvironments, thereby contributing to cancer progression and metastasis. Specifically, EXOs derived from cancer are likely to function as biomarkers for early cancer detection due to the genetic or signaling alterations they transport as payload within the cancer cells of origin. It has been verified that EXOs circulate steadily in bodily secretions and contain a variety of information that indicates the progression of the tumor. However, acquiring molecular information and interactions regarding EXOs has presented significant technical challenges due to their nanoscale nature and high heterogeneity. Colorimetry, surface plasmon resonance (SPR), fluorescence, and Raman scattering are examples of optical techniques utilized to quantify cancer exosomal biomarkers, including lipids, proteins, RNA, and DNA. Many optically active nanoparticles (NPs), predominantly carbon-based, inorganic, organic, and composite-based nanomaterials, have been employed in biosensing technology. The exceptional physical properties exhibited by nanomaterials, including carbon NPs, noble metal NPs, and magnetic NPs, have facilitated significant progress in the development of optical nanobiosensors intended for the detection of EXOs originating from tumors. Following a summary of the biogenesis, biological functions, and biomarker value of known EXOs, this article provides an update on the detection methodologies currently under investigation. In conclusion, we propose some potential enhancements to optical biosensors utilized in detecting EXO, utilizing various NP materials such as silicon NPs, graphene oxide (GO), metal NPs, and quantum dots (QDs).

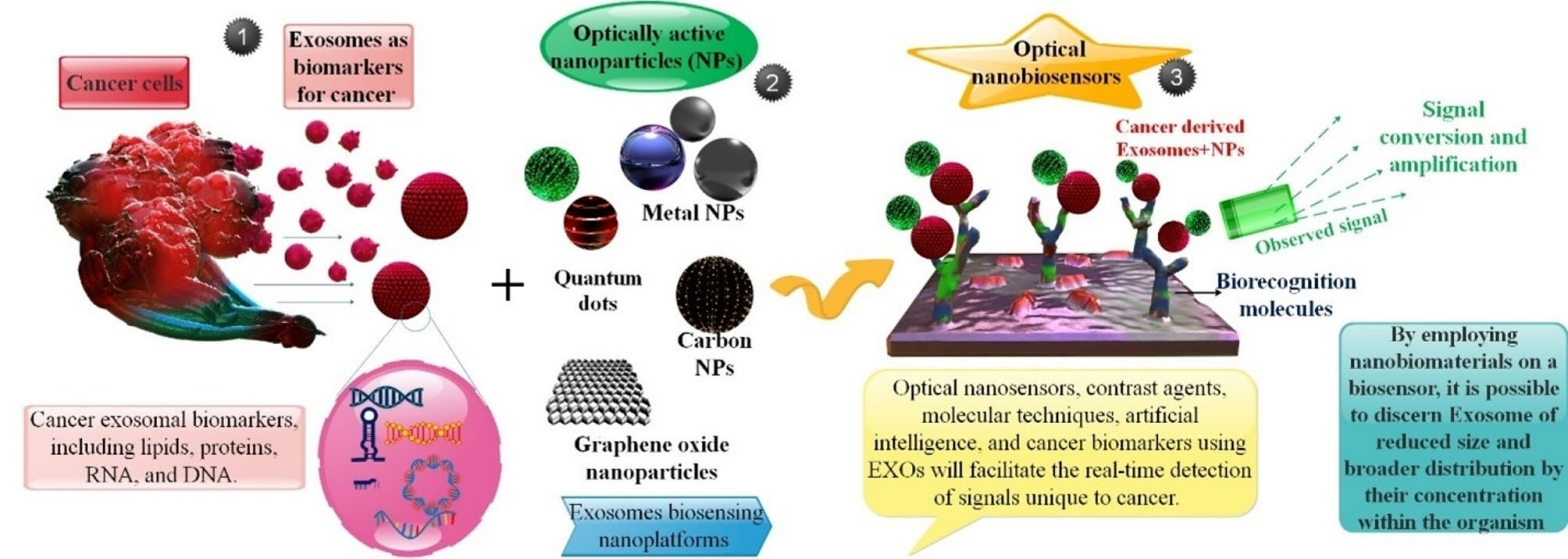

## Introduction

Exosomes (EXOs) are extracellular vesicles (EVs) ranging in size from 30 to 120 nanometers, which originate from endosomes of a multivesicular nature [1, 2]. EXOs are differentiated from microvesicles, which exhibit variability in size ranging from 50 to 1500 nm and are expelled directly from the plasma membrane during budding [3]. EXOs are widely recognized as crucial intercellular communication mediators and contributors to many physiological and pathological processes. Cargo sorting, MVB formation and maturation, transport of multi-vesicular bodies (MVBs), and MVB integration with the plasma membrane are the four essential stages in their biogenesis [4]. Originating in the endocytic pathway are EXOs. An ordinary progression of EXO formation consists of the subsequent stages: (i) an early secretory endosome is formed when the cytoplasmic membrane invaginates; (ii) the payload sprouts inward to form intraluminal vesicles (Ivs) contained within the endosome; (iii) the maturation of late endosomes occurs through acidification; (iv) and finally, extracellular release of ILVs as Evs (EXOs) via fusion with the plasma membrane [5, 6]. They are secreted by the majority of cell types and are expelled into saliva, breast milk, urine, and plasma [7]. The pathophysiology of many illnesses, including cancer, involves EXO-mediated intercellular communication, which is also involved in the control of normal physiological functions [8, 9]. The biology of EXOs is utilized to determine their quantity and integrity precisely. At present, vesicle and particle counts are determined using optical and non-optical techniques, including flow cytometry (FACS), dynamic light scattering (DLS), NP tracking analysis (NTA), and resistance pulse sensing (RPS). Surface plasmon resonance (SPR), transmission electron microscopy (TEM), and resistive pulse sensing (RPS) are non-optical techniques employed in this regard [10].

Global cancer incidence and mortality are increasing at an alarming rate for a variety of reasons, including population aging and expansion [11, 12]. Nevertheless, early cancer detection can improve patient survival rates [12, 13]. An area of inquiry that is relatively new in this discipline is the examination of Evs discharged by cells, where EXOs are advanced. However, due to their diminutive size, EXOs are capable of eluding the phagocyte system and demonstrating their superiority in intercellular communications. EXOs are heterogeneous vesicles derived from membranes that are actively secreted by various cell types [14]. EXOs derived from tumor cells can communicate signals related to tumor metastasis, ascertain the trajectory of cancer cell metastasis, and stimulate angiogenesis and epithelial-mesenchymal transformation (EMT). EXOs derived from tumor cells can alter the migratory status of malignant cells that serve as recipients. EXOs can modulate the tumor microenvironment (TME) through mechanisms such as extracellular receptor signaling pathway stimulation and disruption of cell adhesion [15]. An instance of cancer-induced endothelial cell adaptation via miR-105 takes place during the initial stages of premetastatic niche development. EXO-mediated miR-105 is secreted by metastatic breast cancer cells; it induces tumor cells to metastasize to distal organs by disrupting the barrier function of the endothelial monolayer through its targeting of the tight ligand ZO-1. Increased vascular permeability can potentially facilitate the metastasis and proliferation of malignant cells at a distance. In addition, EXO cargo can enter secondary organs and induce a prometastatic or tumor-supportive phenotype by altering cellular physiology [16]. EXOs, in their capacity as intermediaries of intercellular communication, are increasingly recognized as promising therapeutic and diagnostic agents. Extensive research has identified a multitude of exosomal miRNAs and proteins that exhibit promise as biomarkers in the domains of cancer diagnosis, prognosis, and therapy response prediction [17, 18]. They have been utilized extensively in diagnostics as biomarkers or as part of a collection of biomarkers for disease detection. Consequently, identifying EXOs is vital for advancing EXO research and applications [19].

One innovative way to get around the drawbacks of EXO-based monitoring and diagnostics in the clinic is to use nanosensors. They provide the possibility of multiple marker recognition and real-time tracking. Since nanosensors often include other techniques like microfluidics, electrochemical sensing, and optics, they may also be reasonably priced while maintaining sensitivity. The lack of repeatability in nanosensors is caused by variations in their production from batch to batch. One obstacle that still exists is using EXO-based diagnostics in clinical settings. EXO isolation is labor-intensive and often necessitates expensive kits or apparatus. However, nanodevices could offer an alternative as they provide a sensitive and affordable real-time method of detecting circulating tumor EXOs. These gadgets could also be helpful for platforms that identify many tumors [20]. This paper examines the latest developments in EXO isolation and detection techniques pertaining to the field of (nano)biosensing. Methods of detection that emphasize the optical, electrochemical, and electrical modalities are described in detail. These developments are the foundation for point-of-care testing of cancer and other disorders in the next generation [21]. EXOs have garnered attention as noninvasive and emerging biomarkers in cancer diagnosis. Recently, there has been a proliferation of optical and electrochemical biosensors that aim to detect EXOs with exceptional sensitivity. In response to the growing need for susceptible detection, nanomaterials have been incorporated into diverse methodologies as potent constituents. The unique physicochemical properties, intrinsic biological compatibility, and distinctive catalytic capability of nanomaterials have substantially enhanced the analytical capabilities of EXO biosensors [22]. Specifically, EXOs derived from cancer are likely to function as biomarkers for early cancer detection due to the genetic or signaling alterations they transport as payload within the cancer cells of origin. For the following reasons, EXO-based liquid biopsy merits consideration over conventional tissue biopsy [23]. Generally, cargoes that have been divided into EXOs not only contribute supplementary attributes to aid in their identification but also exhibit potential as biomarkers to diagnose, monitor treatment, and predict prognosis in cancer patients, thereby presenting a novel instrument for liquid biopsy [24]. Biosensors have garnered significant interest in identifying of EXOs owing to their exceptional characteristics, including user-friendly operation, instantaneous output, elevated sensitivity, and extraordinary specificity. These attributes indicate the potential for biomedical applications in the early detection of cancer [25].

Biosensing has been profoundly influenced by an abundance of nanomaterials and nanostructures due to the expansion of nanotechnology. Considerable progress has been achieved in the ability to systematically fabricate nanomaterials exhibiting diverse morphological, chemical, and physicochemical properties. As an illustration, in optical assays, nanomaterials possessing remarkable luminescence characteristics have emerged as a significant substitute for conventional dyes due to their adjustable emission wavelength, high luminescence quantum yield, and favorable photostability. As a result of the intriguing localized SPR phenomenon that is dependent on size and shape, noble metal NPs, particularly silver (Ag) and gold (Au), have been utilized extensively to boost the signal intensity in SPR and SERS assays [26]. For electrode modification, carbon-based nanomaterials (e.g., graphene oxide (GO) and carbon nanotubes (CNTs)) with high electrical conductivity and a high surface-to-volume ratio are always utilized to increase electrode surface area and electron transfer speed. Furthermore, in recent times, there has been significant attention directed towards the photothermal and enzyme-mimetic characteristics of nanomaterials in the context of portable bioassay development. Many signal amplification strategies based on nanomaterials have been devised in conjunction with diverse detection methodologies to enable ultrasensitive identification of biomolecules, such as DNA/RNA, proteins, EXOs, and cells. Enhancing the efficacy of detection methods and improving the capture of EXOs are the two primary objectives of EXO detection. In contrast, magnetic beads (MBs), which have traditionally been employed to bind antibodies or aptamers and selectively capture EXOs from clinical samples, have become more prevalent in the former case [27]. Since the advent of high-resolution optical microscopies and the advancement of modern optical technology, it is now possible to observe the micro/nanostructures of EXOs that were previously impossible to discern optically. Specific sources state that the process consists of only two steps—capture and detection—namely, the direct acquisition of EXOs in bodily fluids like blood and urine. As capture ligands, antibodies and aptamers that target exosomal surface-specific proteins are frequently employed to encapsulate EXOs. These ligands are typically modified to adhere to microfluidic chips, MBs, Au nanostructures, and other substrate materials. EXOs from tumors can be captured exclusively by the specific interaction (e.g., physical interaction, antibody/antigen interaction) between capture probes and EXOs. EXOs could subsequently be identified via microfluidic chip, fluorescence (including Fluorescence correlation microscopy (FCM)), surface-enhanced Raman scattering (SERS), SPR (including LSPR), or colorimetry [28]. Hence, by employing nanobiomaterials on a biosensor, it is possible to discern EXOs of reduced size and broader distribution by their concentration within the organism [29].

We discussed current developments in the detection of exosomal cancer biomarkers utilizing a variety of optical biosensors based on NPs, including QD, GO, AuNPs, AgNPs, Magnetic NPs, and Silicon NPs, in this paper. Furthermore, a summary of each optical nanobiosensors’ benefits and drawbacks is provided as a means of identifying cancer EXOs at the clinical stage. The last section of the report also discusses the landscape and prospects for optical nanobiosensors and optical NPs in identifying exosomal cancer biomarkers. Our goal is to present a novel, all-encompassing optical biosensor concept based on diverse NP forms for identifying EXO biomarkers of all forms of cancer. But, more significantly, we want to explore the drawbacks, restrictions, and advantages of this approach, as well as devise novel approaches to address the obstacles that may arise in the future when using EXO detection in cancer clinical settings.

## EXO biogenesis

EXOs comprise a diverse array of biomolecules, including proteins, cytosolic DNA, RNA, mRNA, lipids, metabolites, and mRNA, and they serve vital functions in intercellular communications [30]. They may protrude directly from plasma membranes or be secreted when multivesicular endosomes fuse with the cell surface. Membrane formation is the first of four primary steps in EXO biogenesis. The other three are endocytosis, multivesicular body formation (MVB development), and sorting, which involves secretion, destruction, and recycling [31]. Strict regulation governs the activation of cell-specific receptors and the signaling pathways that initiate EXO biogenesis [2]. To begin the process of early endosome (EE) development, primary endocytic vesicles must fuse [32]. Many endocytic particles that arrive share their contents and membrane composition through the combination of EEs via independent or clathrin- or caveolin-dependent pathways [33]. There are two possible transformations that EEs go through. One is to become “recycling endosomes,” which return the cargo to the plasma membrane. The other is to transform into “late endosomes,” also known as MVBs. In the plasma membrane, Rab5 and its effector VPS34/p150 play a crucial regulatory role in converting EV to LE. Inward membrane budding, which causes cargo sequestration and dispersion into vesicles, initiates ILV creation in EEs within a few minutes after recycling cargo to the cell membrane [34].

ILV protein sorting is a tightly regulated process that is occasionally endosomal-sorting complex required for transport (ESCRT)-independent and dependent on the (ESCRT) machinery. Four complexes, designated ESCRT-0, ESCRT-I, ESCRT-II, and ESCRT-III, comprise the ESCRT apparatus. The ESCRT-dependent pathway commences with a critical juncture where cargo delivery occurs, as regulated by the protein ubiquitin (ub) checkpoint. The Ub-dependent pathway involves the participation of every ESCRT subunit. The identification of mono-ubiquitinated proteins is facilitated by ESCRT-0 via an HRS heterodimer and STAM1/2 [35]. HRS is a protein found in the cytosol that forms complexes with others, including Clathrin and Eps15. To meet the ubiquitinated cargo, HRS-recruited-clathrin is helpful [36]. ESCRT-I and ESCRT-II subsequently assemble with ESCRT-0 to form a robust recognition domain that exhibits elevated affinity towards the ubiquitinated substrates situated on the endosomal membrane segment where the protein will ultimately proliferate [37]. At last, the buds are released into the endosome when ESCRT-III converges with the complex to pinch off the membrane [38]. ILVs are presently aimed at facilitating cargo degradation at the lysosome, barring the de-ubiquitination of the cargoes by de-ubiquitylating enzymes (DUBs). The complex’s constituents will ultimately be dissociated by the ATPase VPS4 in conjunction with its co-factor VTA and utilized in the subsequent cycle [39].

**Fig. 1:**
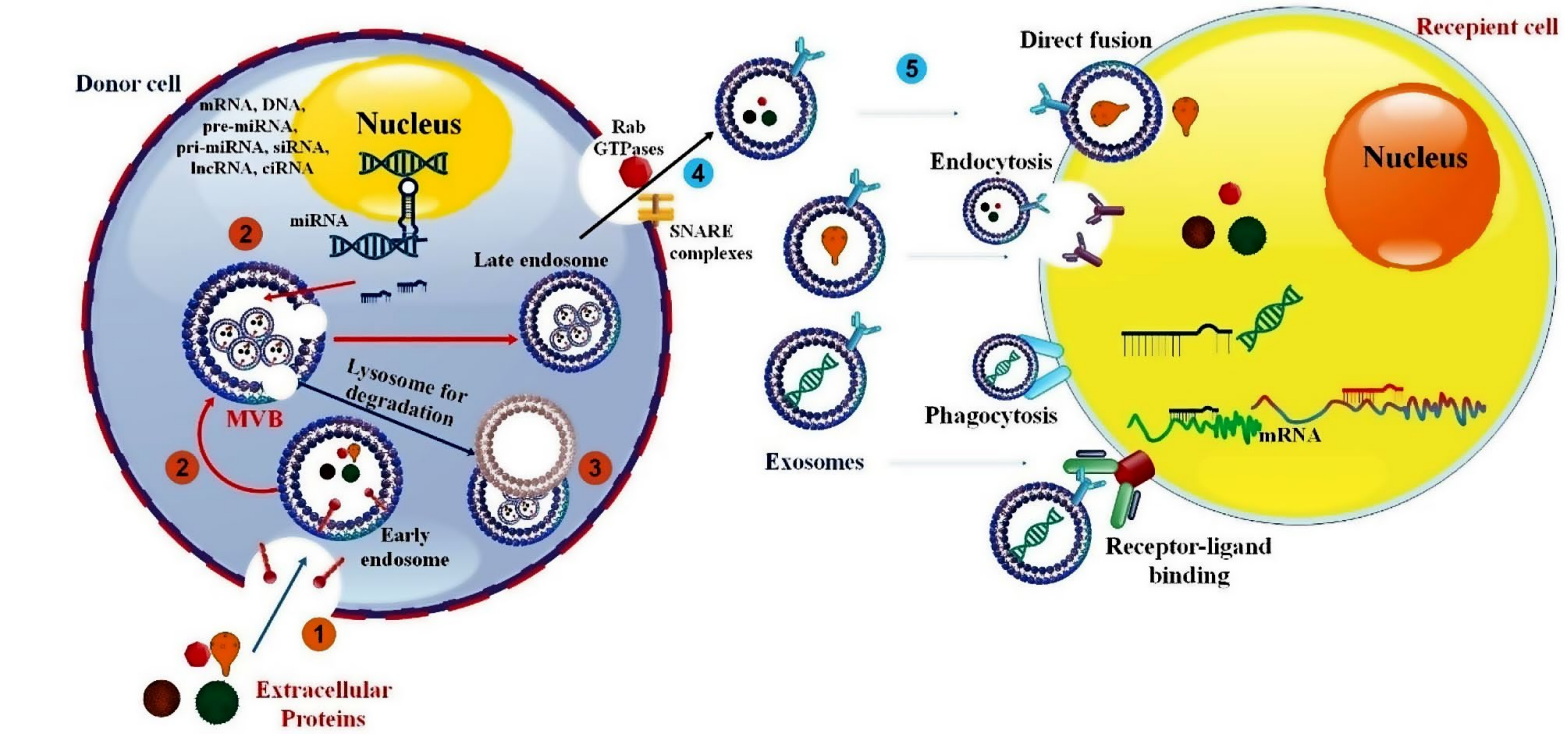
EXO biogenesis and their routes to reach the target cell. The endosomal system is where the biogenesis of EXOs begins. Internalized loads are encapsulated into early endosomes inside the endosomal system, which develop into late endosomes or MVB. Additionally, many substances are moved from the cytosol and perhaps from the trans-Golgi network. Furthermore, MVBs may travel along microtubules to integrate with the cell membrane and release EXOs into the extracellular space, or they can be sent to lysosomes for degradation. The precise process of MVB fusion with the membrane requires several essential components, such as SNARE complexes and Rab GTPases. Three crucial strategies are used by EXOs to effectuate their effects on target cells: (1) recipient cell receptor-mediated signal amplification; (2) direct connection to the plasma membrane and fusion; and (3) endocytosis

Furthermore, various pathways impacting EXO formation have not been identified as dependent on ESCRTs. These pathways include the neutral sphingomyelinase 2-dependent pathway, the heterogeneous nuclear ribonucleoprotein-dependent pathway, the miRNA post-transcriptional 3′end modification pathway, and the RNA-induced silencing complex-related pathway [40, 41]. While the precise mechanism underlying EXO biogenesis remains unknown, multiple recent reports indicate that syntenin and syndecan heparin sulphate proteoglycans may regulate EXO formation [42, 43]. The Rab GTPase pathway controls EXO release. ESCRTs, calcium channels, and cellular pH levels influence the transport and transfer of EXOs to their target cells [44]. Also governed by the silencing of ALIX proteins is the discharge of EXOs [36]. Cargo sorting’s exact mechanism is still a mystery; however, research has pointed to the syndecan-syntenin-ALIX axis as involved in the ESCRT-dependent endo-lysosomal pathway, which is crucial for EXO formation and cargo sorting. Cells that are meant to receive the EXOs either use receptor-mediated endocytosis or undergo receptor-ligand fusion [45] (Fig. 1).

### EXOs in cancer

Cancer-related regulation of EXO biogenesis EXOs facilitates the metastatic potential of cancer cells by enhancing their resistance to chemoresistance, stress tolerance, and evasion of immune surveillance. It is noteworthy that cancer cells employ diverse tactics to progress their disease, including aberrant gene expression, posttranslational modifications, and altered signaling pathways. These strategies govern the biogenesis, composition, and ultimately the functions of EXOs. Significantly, cancer cells can manipulate EXO biogenesis and change the composition and function of EXOs through multiple strategies; this facilitates the release of tumor-promoting EXOs [4]. The role of EXOs as regulators of cancer progression is complex. They can alter the TME to influence nearby cells or cells at specific distant locations, and they often include cancer-derived chemicals in various malignancies. Consequently, they might help detect cancer by acting as a bridge between normal cells and malignant cells. Suetsugu et al. (2013) used green fluorescent protein (GFP)-tagged CD63 to create cell and nude mice models that could see cell-to-cell communication. Cancer cells released labeled EXOs into a culture medium, which were then taken up by other cells. The process begins in the original tumor and continues to the metastatic niche in vivo by the secretion of GFP-EXOs into the surrounding tumor tissue [46, 47]. In the first place, EXOs are indispensable for tumor development and metastasis. EXOs can convey growth-promoting genes, thereby facilitating the proliferation of metastatic cancer cells. For instance, EXOs containing epidermal growth factor receptor (EGFR) promote this pattern for liver-specific metastasis formation [48].

Since EXOs are both non-immunogenic and non-toxic, they may provide a viable option for medication delivery in cancer treatment. Targeted cancer treatment using an EXO-based delivery strategy for paclitaxel and Adriamycin has shown promising results with little immunogenicity and toxicity [49, 50]. In addition, EXOs can be produced by various cell types. Furthermore, EXOs have a greater capacity for permeation through tumor cells than liposomes. One further benefit of EXOs is their ability to deliver medicines specifically to cancer cells by utilizing proteins that enable them to target particular cells and tissues. Moreover, due to their diminutive dimensions, EXOs possess the ability to traverse diverse barriers with relative ease, including the blood-brain barrier [51]. Moreover, EXOs may be simply modified to enhance their ability to target cancer cells. The potential of tumor-derived exosomal RNA as biomarkers in cancer screening and diagnosis is supported by mounting data in various body fluids, including blood [52]. Utilizing bioengineered EXOs, functional RNAs, and anticancer medications have been delivered cell-specifically to cancer cells, including CSCs. EXO-mediated targeting of CSCs is one of the most promising strategies for developing cancer therapies. To generate modified EXOs, specific proteins are engineered into the donor cells to bind to the EXO membrane. Particular producers of CSCs, including CD44, CD24, CD133, and CD200, can be targeted with EXOs [53]. Furthermore, EXOs are very safe and biocompatible. By delivering cellular contents to recipient cells, EXOs are essential in intercellular communication [34]. By transferring bioactive molecules from cancer cells to cells in TME, they could promote the progression and development of cancer [54]. EXOs contain bioactive chemicals that may affect cell proliferation, differentiation, and death signaling pathways when transported to distant or close recipient cells. By playing a role in cancer cell development, invasion, metastasis, angiogenesis, and treatment resistance, EXOs control the biological activities of cancer cells, TME cells, and distant recipient cells [41]. Proteins are among the numerous molecules that EXOs can transport, contingent upon their origins and in vitro culture conditions. EXOs have been demonstrated to contain a diverse array of proteins that perform functions associated with the membrane, such as annexin, Rab GTPase, cellular adhesion proteins (including integrins and tetraspanins), cytoskeletal proteins (including actin and myosin), and heat shock proteins (including Hsp70) [55]. Annexins, RAB5/RAB7, and TSG101 are fusion and membrane transport proteins found in EXOs that have recently been implicated in cancer initiation and progression. For instance, the antitumor immune cells may be activated when EXOs produced by dendritic cells (DCs) contain MHC-I, which binds to peptides produced by tumors [56]. An intriguing investigation has unveiled that EXO-derived membrane surface protein TRAIL is capable of transmitting apoptosis-related signals to tumor cells and thereby inducing apoptosis in said cells. Furthermore, EXOs containing SIRPα can bind to CD47 on tumor cells, stimulating macrophage phagocytosis and ultimately impeding the progression of cancer [57, 58].

**Fig. 2:**
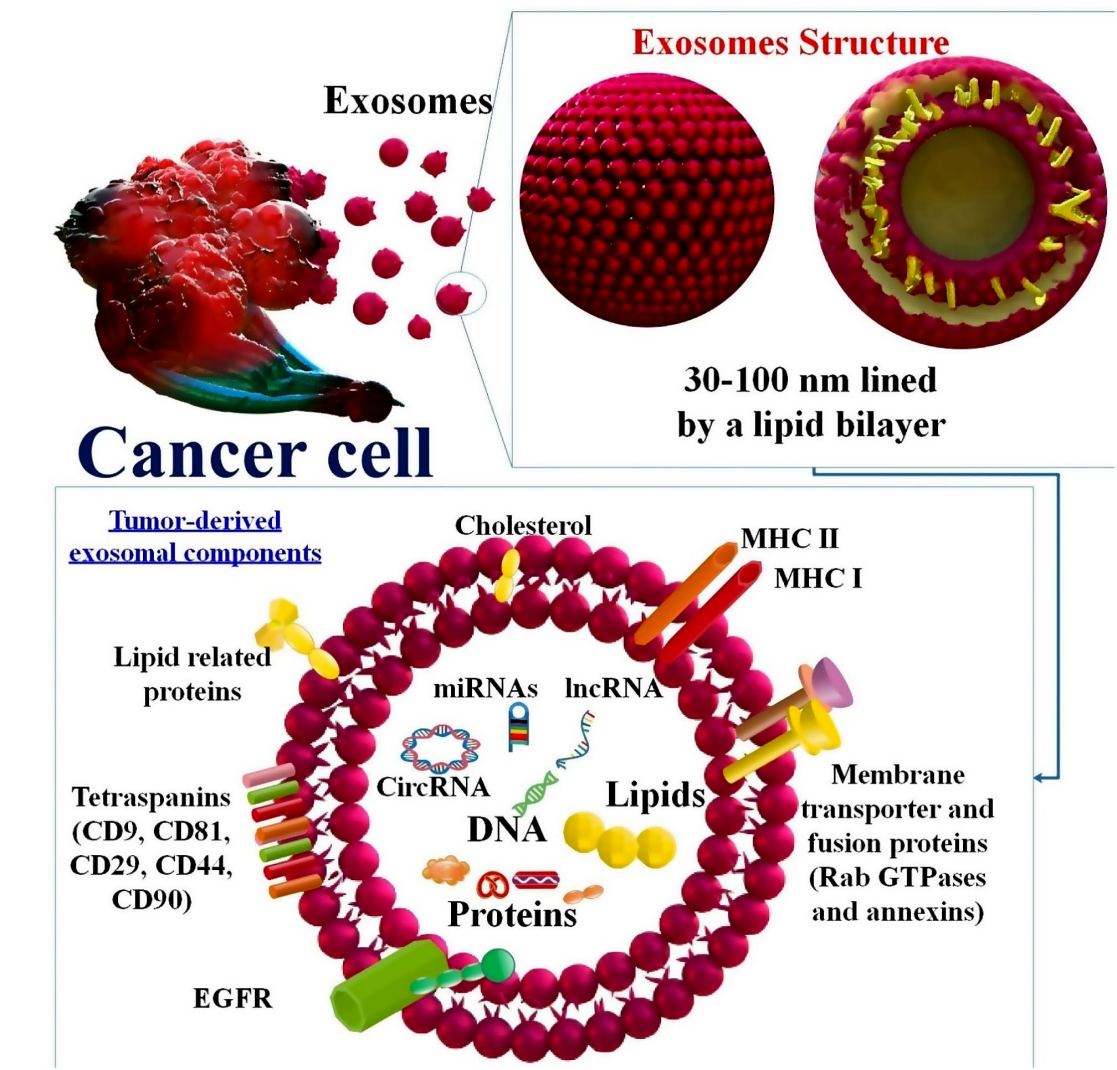
Plasma membrane-derived exosomes (EXOs) are phospholipid bilayers composed of cytoplasmic components originating from the progenitor cell. The composition of EXOs is influenced by the parent cell’s health status, the type of cell from which they originated, and the existence or absence of extracellular stimuli. Many EXOs exhibit homology in terms of proteins, lipids, and microRNAs [60]

In addition to acting as messengers in intracellular communication, cancer-derived EXOs (CDE) can modify nearby and faraway microenvironments. Also, unlike liposomes, EXOs may quickly pass through tumor cells. An increase in the efficacy of EXOs in targeting cancer cells is being pursued. EXOs also have the added benefit of not being too big, which allows them to bypass most of the typical obstacles to cell penetration. Tissue-derived EXOs (TDEs) facilitate drug resistance, angiogenesis, invasion, progression, and proliferation of cancer using intercellular communication within the TME. EXOs are exchanged between cancer cells and other tissues; they serve to establish a premetastatic niche, elude immune surveillance, and impart resistance to multiple drugs. Although EXOs present in the circulation are promising early detection indicators for cancer patients, their efficacy as clinical biomarkers is constrained by their heterogeneity and diverse origins [59] (Fig. 2).

## Biomarkers are used to confirm EXOs

**Fig. 3:**
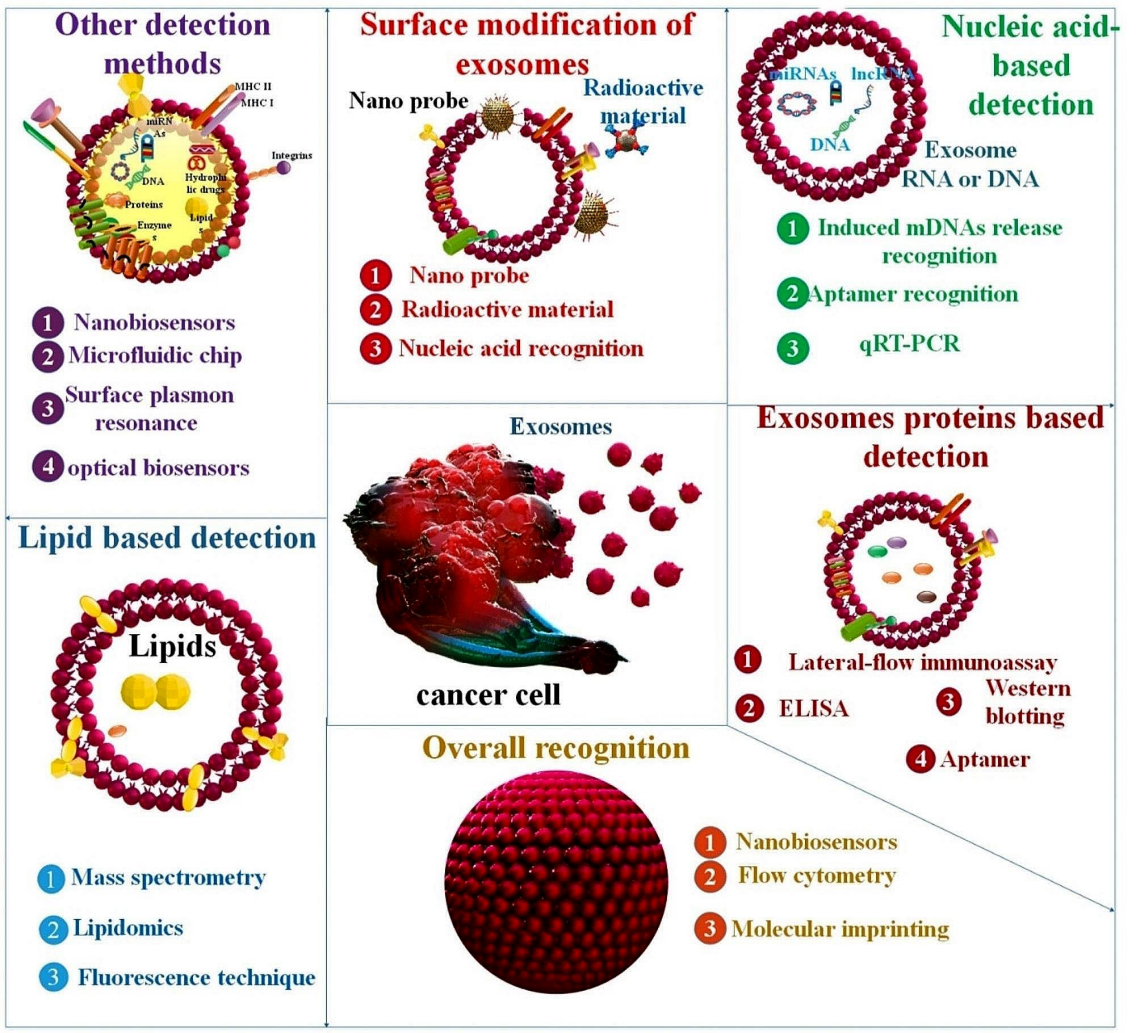
The dynamic active loads that makeup EXOs are diverse and include DNA, RNA (including miRNAs), lipids, enzymes, and proteins. Their importance in identifying biomarkers for clinical diagnostics is universally recognized since they convey proteins and nucleic acids from recipient cells that indicate pathophysiological conditions. The presence of EXOs carrying nucleic acid or protein biomarkers may be detected utilizing microarray analysis, DNA sequencing, ELISA, or polymerase chain reaction. Plus, several biosensors have been developed to detect exosomal biomarkers including proteins, nucleic acids, and EXOs produced by tumors

Research relies heavily on the EXO’s properties, its separation ability, and its quantitative detection [61]. Numerous detection methodologies rely on biomolecule recognition, including nucleic acids, proteins, and lipids [62]. Nine and Llorente, as well as Rajput et al., enlist numerous lipids, protein molecules, and nucleic acids linked with EXO synthesis and release as a biomarker, allowing for simple detection. EXO detection techniques include optical, electrochemical, immunoreaction, aptamer-based, fluorescence, SPR, SERS, chromatography, and microfluidic detection approaches. One or more detection techniques have also been integrated to improve the overall efficiency of the operation [63, 64] (Fig. 3).

### Nucleic acid-based detection

These nucleic acid molecules are free to communicate and convey information between cells in the body, which is frequently associated with illnesses (especially in identifying cancers). This opens the door to RNA-based cancer medication treatment applications [65]. To identify nucleic acids, electrochemical assays and next-generation sequencing are commonly employed. Because the genetic materials (DNA/RNA) are predominantly contained within the EXO, they continue to be encased in the lipid bilayer, complicating diagnostic procedures involving nucleic acid recognition. Before identification, nucleic acids are released to circumvent this. A technique was devised by Zhang et al. in which they utilized an adaptor magnetic bead as a bioconjugate to stimulate the liberation of multiple mitochondrial DNAs (mDNAs) from LNCaP cells. The released mDNAs underwent hybridization with the Au electrode-immobilized probe DNAs. The electrochemical detection of these mDNAs suggested the existence of EXOs affiliated with the tumor [66]. Tan et al. developed an aptamer employing electrochemiluminescence (ECL) to identify malignant EXOs from hepatocyte cells. Their method involved the examination of DNA nanostructure and nano-tetrahedron. It was ascertained that the maximum low limit of detection (LOD) of the employed aptasensor system was 3.96 × 10^5^/mL. Sun et al. investigated the application of next-generation sequencing to detect nucleic acids in a separate investigation. Utilizing next-generation sequencing, the miRNA expression profile of bovine milk was analyzed [67, 68]. The creation of very sensitive probes is crucial for the simultaneous identification of multiplexed cancer-associated nucleic acids. Silver-containing bimetallic NPs have the potential to be used as SERS nanoprobes for disease diagnosis since they can provide robust and consistent signals. The direct synthesis of such SERS nanoprobes is still quite difficult, however. Investigators describe the effective synthesis of Au-Ag nano snowmen that are produced via a DNA-mediated method and connected to Raman dyes that contain thiol. To detect the target genes jointly boosted by the SERS nanoprobes in a sandwich hybridization experiment, stable SERS-enhanced Au substrates are also created and employed as enriching containers. This indicates that the target gene may be detected with a LOD that is almost 0.839 fM. These doubly-enhanced SERS nanosensors are also used to concurrently identify, with excellent sensitivity and specificity, the three kinds of genes linked to prostate cancer while also demonstrating a solid resistance to disruption in real-world samples. Multiplexed simultaneous detection of cancer-related genes may open new possibilities for biological applications [69].

Tian et al. described a straightforward method for using SERS nanoprobes to find EXOs. EXOs produced from the HepG2 hepatocellular carcinoma cell line are used in the research as model analytes for the diagnosis of liver cancer. The SERS probe was Au nanostars modified with a bivalent DNA anchor tagged with cholesterol. The target EXOs were first collected by immuno-magnetic beads, and the trapped EXOs were then labeled with the SERS nanoprobe via a hydrophobic contact between the lipid and cholesterol membranes, creating a sandwich complex. The resultant immunocomplexes might be placed on a silica slide for detection and then magnetically collected. The EXO concentration and associated SERS signal in this investigation showed a linear relationship, with the sensor reaching a detection limit of 27 particles/µL. The SERS signal ranged from 40 to 4 × 10^7^ particles/µL [70].

A PIA biosensor that can accurately and instantly identify EGFR + EXOs was described by Zeng et al. To allow portable and sensitive detection for early cancer diagnosis, the sensor may be coupled with a smartphone or used with a miniature microscope. EGFR was used as a lung cancer biomarker in this investigation. The detection limit was achieved with a desk-top optical microscope and a smartphone-based microscope, measuring 3.86 × 10^8^ and 9.72 × 10^9^ EXOs /mL, respectively. A desk-top optical microscope might further lower the detection limit [71, 72].

In an additional investigation, scholars present novel findings regarding the utilization of their internally designed Localized Surface Plasmon Resonance biosensor with self-assembly Au nanoislands (SAM-AuNIs) to differentiate EXOs from MVs isolated from A-549 cells, SH-SY5Y cells, blood serum, and urine derived from a mouse model of lung cancer. In contrast to MVs, EXOs exhibited a discernible response to the unmodified localized surface plasmon resonance (LSPR) biosensor, indicating that EXOs and MVs engage in a distinct biophysical interaction with SAM AuNIs. The sensor demonstrates a linear dynamic range of 0.194–100 µg/ml and achieves a LOD of 0.194 µg/ml. This finding not only elucidates the unique membrane characteristics of tumor-derived EXOs and MVs but also supports the design of innovative long-wavelength solid-phase reflector (LSPR) biosensors that enable direct identification and segregation of heterogeneous EVs [73].

The biosensor based on SERS demonstrates sensitive detection. But the process of preparing the sample—enriching EXOs using magnetic beads—takes more than ten hours. SAM-AuNIs are used by the LSPR biosensor to facilitate EXO separation. On the other hand, non-specific binding noise poses a severe problem to clinical applications. Some sensors use enzymatic amplification and magnetic enrichment to provide substantial dynamic ranges and detection limits. These methods will probably have to be included in the sensing procedure to facilitate the quick and precise identification and enrichment of relevant biomarkers in intricate clinical samples. Future biosensors should have improved sensitivity and specificity as well as speed, usability, and affordability [72].

### Proteins based detection

Prior studies identified numerous specific proteins expressed on the surface of EXOs, a characteristic that sets EXOs apart from other vesicles. The aforementioned proteins consist of surface growth factor receptors (EGFR), tetraspanins (CD9, CD82, CD81, and CD63), heat shock proteins (Hsp 60, Hsp 70, Hsp 90), and synthetic proteins (TSG, Alix). Furthermore, tetraspanins exhibit a substantial enrichment on the EXO surface, rendering them optimal markers for the identification and quantitative analysis of EXOs [61]. Consequently, immune recognition of proteins for EXO detection is one of the most prevalent techniques in identification. A multitude of EXO analytical methodologies have been developed by scientists through the integration of protein immunorecognition technology, electrochemical analysis, chromatographic analysis, SERS, and microfluidic detection platforms [74, 75].

Protein immunorecognition technology has been implemented in numerous EXO analytical procedures recently. Yu et al. devised a field-effect transistor biosensor that employs antibody-modified reduced GO to precisely detect the CD63 protein present on EXOs for quantitative EXO analysis. This technique demonstrated a low detection limit of 33 particles/µL and a high specificity [76]. Wang et al. documented an AuNP -amplified surface acoustic wave sensor that demonstrated exceptional sensitivity in the identification of EXOs. The carboxyl group was generated via the self-assembly of thioglycolic acid via an Au-S bond. Anti-CD63 antibodies were immobilized onto the chip to facilitate the targeted identification of EXOs. Secondary EpCAM antibodies were employed for amplification and recognition. In conjunction with Au NPs, a surface acoustic wave (SAW) sensor was developed to detect the presence of EXOs in the blood samples of cancer patients. The sensor can detect a minimum of 1.1 × 10^3^ EXOs per microliter [77].

Furthermore, aptamers are oligonucleotides chosen via the exponential enrichment of ligands (SELEX) method of systematic evolution. They possess fundamental qualities such as flexibility in the nucleic acid chain and spatial conformational diversity, among others. Proteins establish particular and favorable bindings with aptamers through various mechanisms, including superposition, complementary geometry, electrostatic, and hydrogen bonding [78]. Nucleic acid aptamers possess several advantages over antibodies, including their facile synthesis, low cost, excellent long-term stability, and capacity for chemical modification. EXO surface marker protein aptamers have been utilized in numerous studies over the past few years to accomplish specific EXO recognition and detection [63]. An ECL sensor for EXOs and their surface proteins was developed by Zhang et al. utilizing Au NPs that had been modified with Ti_3_C_2_ and CD63 aptamer. This approach not only facilitated the precise and efficient recognition of EXOs but also furnished barecatalytic surfaces of Au NPs exhibiting significant electrocatalytic activity. Additional protein aptamers were utilized by some analytical techniques to detect EXOs [79].

EXOs released by cancer that contain specific surface proteins are regarded as novel liquid biopsy biomarkers with high diagnostic value. Despite this, the scarcity of EXO surface proteins continues complicating the development of sensitive and rapid quantitative methods for their quantification. A novel platform was devised in this study, employing an integrated magneto-fluorescent EXO (iMFEX) nanosensor to facilitate the swift and precise identification of EXOs originating from malignant cells. Before capturing EXOs, magnetic NPs were coated with a DNA tetrahedral lipid probe for enhanced efficiency. By initiating catalytic hairpin assembly and explicitly recognizing the EXOs PD-L1 protein, the bifunctional aptamer that had been rationally designed converted protein signals into H1/H2 duplexes. In conclusion, the Cas12a protein trans-cleavage reporter FAM-TTATT-BHQ1 was stimulated for fluorescence signal amplification by the H1/H2 duplexes. The practical detection limit of the proposed iMFEX nanosensor was 1.71 × 10^3^ particles/µL, and it demonstrated exceptional sensitivity and specificity toward PD-L1 positive EXOs (range: 2.86 × 10^3^ to 2.86 × 10^7^ particles/µL) by integrating fluorescence signal amplification and magnetic separation. By employing the iMFEX nanosensor, researchers successfully monitored the dynamic fluctuations in exosomal PD-L1 expression triggered by reagents and distinguished non-small cell lung cancer patients from healthy individuals, thereby establishing the clinical applicability potential of the platform [80].

Graphene field effect transistors (GFETs)-based biosensors may make it possible to create point-of-care diagnostic instruments for the early identification of disease stages. However, problems with graphene sensor manufacturing yields and repeatability, as well as undesired species detection and Debye screening, have hindered the broader clinical use of graphene technology. Here, researchers show that significant therapeutic outcomes are possible with this wafer-scalable GFETs array device. They provide a robust and accurate portable GFET array biosensor technology for the 45-minute detection of pancreatic ductal adenocarcinoma (PDAC) in patient plasma using particular EXOs (GPC-1 expression). This case study has excellent therapeutic value. Investigators improved the analytical performance of GFET biosensors by using an internal control channel and creating an optimal test strategy to enable repeatable detection in blood plasma. Using samples from eight healthy controls and eight PDAC patients, the GFET biosensor arrays can distinguish between the two groups and identify early stages of cancer, including stages 1 and 2. In addition, Researchers verified that GPC-1 was expressed at a greater level and discovered that the concentration in PDAC plasma was, on average, more than a factor of ten higher than in healthy samples. Researchers found that these features of GPC-1 malignant EXOs are in charge of increasing the number of target EXOs on graphene’s surface, which enhances the GFET biosensors’ signal responsiveness. The creation of an accurate instrument for the prompt identification of pancreatic cancer seems to be very promising with the use of this GFET biosensor platform [81].

### Lipid-based detection

Due to the presence of lipid bilayers, which contribute to the inclusion stability of EXOs and comprise their outermost portion, which is also abundant in cholesterol, phospholipids, and polyglycerol, several aptamer-based methods have been devised to selectively target these lipid components to facilitate the detection of EXOs. This has resulted in a substantial decrease in interference signals and guaranteed EXO detection with high specificity and sensitivity. In the context of EXO detection and identification, lipids may also bind to the surface protein of the EXOs; this phenomenon is called “double marker recognition.” Cholesterol, for example, is employed as a reagent to detect the binding of a target with aptamers or surface proteins such as CD63. In some instances, cholesterol is conjugated with a magnetic separation technique, which multiplies the method’s sensitivity by a significant amount [82, 83]. Multiple lipid molecules (found in EXOs) were identified and extracted from urine samples of individuals who have prostate cancer in a research investigation conducted by Skotland et al. Additionally, various lipids were quantified utilizing techniques such as mass spectrometry (MS) and lipidomics [84]. To ensure the preservation of the constituents housed within EXOs, a lipid bilayer comprises the outer EXO layer. The precise identification and detection of EXOs were accomplished by utilizing lipid components, including but not limited to cholesterol, polyglycerol, and phospholipids. Tian et al. created a high-sensitivity microchip platform for EXO detection in conjunction with integrated nucleic acid amplification microchip digital detection by affixing DNA oligonucleotide binding to the lipid double layer of EXOs. Lipid components are prevalent on the surfaces of EXOs [85]. To identify EXOs in urine samples, the cholesterin nanoprobe was anchored during the detection process. Yao et al. developed a fluorescence technique for detecting EXOs by enhancing the signal of copper NPs. EXOs were separated magnetically utilizing magnetic NPs modified with cholesterol, which took advantage of the hydrophobic interaction between cholesterol molecules and lipid membranes. This technique is straightforward and rapid, enabling detection to be concluded in 2 h [86].

Furthermore, the identification and detection of EXOs can be facilitated by combining lipid recognition and surface-specific proteins. Recognition of two markers was implemented. Combining aptamers that recognize CD63 on the surface of EXOs with magnetic bead separation technology and cholesterol probes that target lipid layer localization are among these. Ultimately, a sensitized and precise EXO detection strategy has been developed [82]. Zhang et al. performed quantitative analysis of EXOs by visual detection or UV-vis spectrophotometer using the amplification of the hybrid chain reaction (HCR) signal of alkaline phosphatase (ALP), in conjunction with a cholesterol-modified DNA probe and CD63 aptamer magnetic bead marker capture, for lipid identification in EXOs. This approach is straightforward and precise, making it simple to execute. The lipid bilayer structure of EXO surfaces is frequently employed in conjunction with other recognition and detection methods. This technique significantly reduces interference signals, ensures high sensitivity, and achieves a high degree of specificity in EXO detection. However, this technique is more expensive and entails a more complicated modification and operation process [87].

Researchers demonstrated the ESCRT-independent lipid-based mechanism that contributes to the formation of MVB is a critical step in EXO biogenesis. n-SMase, a crucial enzyme in lipid metabolism, facilitates the conversion of sphingomyelins (SMs) to ceramides (Cers) via hydrolysis, thus encouraging the development of ILVs within MVBs. Consequently, the modulation of EXO release can be achieved by regulating n-SMase. Lipid extracts in EXOs from healthy volunteers, HCC, and ICC patients were analyzed using a novel pseudotargeted lipidomics method focusing on sphingolipids (SLs). This was done because cancer-associated cells release a greater quantity of EXOs compared to healthy cells. The objective was to determine whether cancer-related characteristics regulate the release of EXOs via the pathway mentioned above. A multivariate analysis utilizing SLs expression effectively differentiated three groups, suggesting that the SLs expression varied among the three groups. Within the cancer groups, there was an up-regulation of two species of critical Cers, designated Cer (d18:1_16:0) and Cer (d18:1_18:0), but a down-regulation was observed in 55 types of SLs, including 40 species of SMs (d18:1_16:0), SM (d18:1_18:1), and SM (d18:1_24:0). Conversely, several SM/Cer species demonstrated substantial down-regulation. The significant augmentation of the SMs hydrolysis to Cers process observed during EXO biogenesis implies that characteristics associated with cancer might facilitate an expansion of EXO release via a mechanism unrelated to ESCRT. Furthermore, differential SLs possess the potential to serve as biomarkers for disease diagnosis and classification, as evidenced by their AUC values of 0.9884 and 0.9806, respectively, when comparing healthy groups to HCC and ICC groups. Furthermore, an association analysis was performed on the cell lines. It revealed a negative correlation between changes in the SM/Cer contents of cells and their EXOs and the levels of released EXOs. This suggested that n-SMase and subsequent SL expression could be modulated to regulate EXO release levels [88].

Ultra-high-resolution Fourier transform mass spectrometry (UHR-FTMS) has been used by researchers to examine the lipid profiles of blood plasma EXOs to identify non-small cell lung cancers (NSCLC) early on. EXOs are nanovehicles that are secreted by a variety of cells and tumor tissues that trigger critical biological processes including immune system regulation and tumor growth. 91 NSCLC participants (44 early stage and 47 late stage) and 39 normal subjects had their plasma exosomal lipid profiles obtained. To categorize the data, researchers used the multivariate statistical techniques of Random Forest (RF) and Least Absolute Shrinkage and Selection Operator (LASSO). To choose the top 16 lipids using the RF technique, the allocated lipids’ Gini relevance was computed. Seven characteristics were selected using the LASSO technique by a grouped LASSO penalty. Using the chosen lipid characteristics, the Area Under the Receiver Operating Characteristic curve was 0.85 and 0.88 for RF and 0.79 and 0.77 for LASSO, respectively, for early and late-stage cancer compared to normal people. These findings highlight the usefulness of LASSO and RF for developing biomarkers based on metabolomics data, as they provide reliable and independent classifiers for sparse data sets. Lipid characteristics that effectively differentiate early-stage lung cancer patients from healthy people are identified using LASSO and Random Forests [89].

## EXOs detection methods

Increasing interest in electric vehicles has prompted scientists to develop suitable methods for quantifying them. ELISA is a potent instrument in this regard, as using tetraspanins provides exceptional specificity in identifying and quantifying particular subsets of EVs, including EXOs. Additionally, their dimension and the quantity of EVs could be approximated using TEM. ELISA is preferable, provided that the relevant standard values are utilized. While it is not possible to determine the overall concentration of EVs due to their heterogeneity and the presence of subtypes with distinct membrane proteins that serve as markers, this technique can identify and quantify EVs originating from a particular cellular source. CD24 and EpCAM expression can be used to identify EXOs derived from ovarian cancer cells, demonstrating the potential utility of EVs in cancer diagnostics [90]. Furthermore, the prospective biomedical applications of the ELISA method for detecting EV derived from various sources (cell culture and biological fluids including plasma, serum, urine, and cerebrospinal fluid) are made possible by its adaptability for optimization. When the volume is adequate, it is possible to analyze the same sample multiple times to investigate various targets of interest. Potentially valuable for biomedical research, the ELISA method’s adaptability for optimization permits its utilization in detecting EV originating from multiple sources (cell culture and biological fluids including plasma, serum, urine, and cerebrospinal fluid) [91].

Western blotting, alternatively referred to as immunoblotting, is a method that separates and visualizes proteins using gel electrophoresis, contingent upon the characteristics of the sample or the gel, after the application of particular antibodies. In EV research, this method is commonly utilized to ascertain the existence of purified EXOs via their specific surface proteins. Proteins in a mixture are segregated by type and molecular weight using gel electrophoresis. After these substances are transferred to a membrane, a band is formed by each protein [92].

The utilization of NTA to ascertain particle size distribution and concentration is growing in popularity. It is based on the individual detection of particles and the tracing of their Brownian displacements using a charge-coupled device (CCD) camera to record the dispersed light of a laser beam. NTA can identify and analyze particles in real-time within an approximate range of 10–20 nm to 1000–2000 nm. The lower LOD is primarily determined by the optics of the instrument and the quantity of light scattered by the particles. NTA applies to samples that are both monodisperse and polydisperse. Consequently, various subtypes of EVs can be classified utilizing this system. In addition, NTA measures additional parameters, including particle concentration, zeta potential, and the relative intensity of light scattered [93]. Biomedical applications are possible for NTA, which is among the most extensively used techniques for analyzing the concentration and size distribution of EVs. Varying sizes of EVs may be observed under pathological conditions. Indeed, the NTA system identified elevated levels of EV in patients diagnosed with prostate cancer or breast cancer when compared to a control group of healthy individuals. EV levels were recently discovered to be elevated in a rodent model of Parkinson’s disease [91, 94, 95]. Phenotyping of particular types of EV may be performed by labeling fluorophore-conjugated antibodies, which is not feasible with conventional FACS. Despite all these advantages, current methods of EV isolation cannot separate EVs according to their size. NTA allows size characterization but not their separation. In this sense, asymmetrical-flow field-flow fractionation (AF4) is a technique that enables EV fractionation according to their size. This technique is relatively new in the field of EV study, yet it requires further work on standardization for their analysis to achieve reproducible results [96, 97].

The lateral-flow immunoassay (LFIA) is a widely recognized point-of-care diagnostic technology that provides several benefits, including enhanced diagnostic accessibility, cost reduction, expedited analysis times, and user-friendliness [98, 99]. LFIA is capable of detecting these vesicles due to the protein content on their surface, as was previously mentioned about ELISA. Particularly abundant in the membrane of EXOs, tertracaines are frequently employed as EXO biomarkers owing to their broad distribution across nearly all cell types. By combining antibodies that target these proteins, LFIAs for the detection of EXOs from various sources can be created. Due to their diminutive dimensions, EXOs can traverse the membrane without impeding the flow or obstructing the pores until they are captured by the antibody that is immobilized on the detection line. Additionally, the LFIA platform has the potential to be expanded into a multiple-target assay through the utilization of distinct antibodies on various test lines. This permits the identification of a wide variety of EVs according to the protein content of their surfaces. EV measurement can be accomplished using a calibration curve, as in ELISA. The variation in protein composition, density, and localization of tetraspanins at the EXO surface can have a significant impact on the LFIA detection of EV. Consequently, it is essential to develop specialized LFIAs for particular EXO subpopulations [100–102].

## Biosensors and nanobiosensors for EXO detection

Biosensors and nanobiosensors for detecting EXOs Biological receptors or interactions are employed to achieve a remarkable degree of specificity in identifying the unique target biomarker, as opposed to non-specific molecules present in medical samples [21]. In the past few decades, biosensors have been extensively utilized in medical and biological markets across the globe as dependable, rapid, and accurate analytical techniques [103, 104]. Pregnancy tests and portable glucometers printed on paper are the most effective commercial biosensors. Biosensors are analytical instruments specifically engineered to detect and/or quantify biomarkers accurately. Biological receptors or interactions are employed to achieve a remarkable degree of specificity in identifying the unique target biomarker, as opposed to non-specific molecules present in medical samples [105]. The transducer, being an additional critical component of a biosensor, converts biological signals into electrical or optical signals that are quantifiable. Based on their transducer types—mechanical, electrical, optical, electrochemical, or thermal—biosensors can be categorized. Each variety has advantages and disadvantages, and researchers and developers select one according to their specific requirements and designs [106, 107]. As a result of the development of nanotechnology, most biosensors are incorporating various nanomaterials to improve detection sensitivity and precision [108]. These constitute the majority of biosensors on the market today and are composed of nanomaterials in various compositions, shapes, sizes, and source materials. They are simply referred to as nanobiosensors. These advancements are progressively substantial and provide substantial improvements for developing biosensors [109]. Due to their substantial specific surface area, NPs facilitate the efficient attachment of EXOs. In contemporary times, there is a heightened focus on magnetic nanoplatforms and nano-biosensors as viable means to identify and capture EXOs, owing to their facile segregation via magnetic fields. Furthermore, target specificity can be imparted by conjugating these nanomaterials with ligands that are specific to the target. On the contrary, NPs might lack surface functional groups that enable conjugation with other entities, such as antibodies or aptamers. In pursuit of this objective, surface functionalization or modification of NP is executed [110–113].

Recently, biological and nanobiosensor technologies have been investigated to detect and quantify EXOs. Optical, electrochemical, and electrical techniques comprise the majority of EXO biosensor development sensing mechanisms [114]. Most studies have employed nanomaterials to enhance the precision and sensitivity of biosensors to detect minute quantities of EXOs [115]. To improve overall performance, additional strategies, such as microfluidic and chip-based devices, have been implemented in some instances [116]. As of now, electrochemical nanosensors have emerged as highly significant tools in detecting EXOs due to their straightforward operation, exceptional accuracy, and dependable repeatability [117]. Enrichment monitoring Practical applications are becoming increasingly interested in integrated biosensors for EXO analysis due to their rapid, cost-effective, and ultrasensitive sensing capabilities. Furthermore, these nascent methodologies leverage biomolecular probes, NPs, magnetic materials, immune sandwiches, optical spectra, and microchips to construct integrated analytical platforms that represent significant advancements in property profiling [5]. This feature describes the latest developments in integrated technology-based EXO profiling, with a focus on (i) the underlying principles of these kinds of emerging biosensors for EXO detection, (ii) sophisticated techniques developed for EXO profiling, such as field-effect transistor (FET), electrochemical, and SERS methods, along with typical examples; and (iii) the opportunities and current difficulties of developing EXO profiling systems [19]. Surface-enhanced Raman spectrometry, fluorescence, microfluidics, and other techniques have been suggested to enhance sensitivity, streamline the procedure, or diminish the expense associated with EXO detection in real-time monitoring. Disturbances in the complex matrix, however, necessitate the use of apparatus and relatively costly sample pre-treatment for most of these techniques. Consequently, their speed and sensitivity are insufficient for point-of-care (POC) detection of low-abundance EXOs in clinical samples [118]. Biosensors, on the other hand, have drawn a lot of interest in identifying EXOs because of their superior characteristics, which include easy operation, real-time readout, high sensitivity, and amazing specificity. These attributes point to potential biomedical applications in the early detection of cancer [25].

Surfaces undergo the formation of various self-assembled monolayers (SAMs), which not only contribute functional groups but also modify surface energy [119]. Reagents like silane or thoil are utilized for this. For instance, the -NH2 and -COOH groups are provided on surfaces by 3-aminopropyltriethoxysilane (APTES) and octadecyltriethoxysilane (OTES), respectively. Ligands may be bonded to surfaces directly via SAMs. To effectively detect EXOs, linker molecules such as glutaraldehyde or 1-ethyl-3-(3-dimethylaminopropyl) carbodiimide (EDC)/N-hydroxysuccinimide (NHS) are used to conjugate target-specific entities. The biomacromolecules found in EXOs are used by the built nanoplatforms as a means of detection. Detections based on lipids and proteins are often combined. Back et al. used a multifunctional CaTiO_3_:Eu^3+^@Fe_3_O_4_ nanocomposite to establish a quick, easy, and effective method for isolating and detecting EXOs. High-energy ball milling was used to create the nanocomposite. The hydrophilic phosphate head group of exosomal phospholipids bound to CaTiO_3_:Eu^3+^@Fe_3_O_4_ to form EXOs. Furthermore, the target antigen (CD81) was found using an immunological test based on SERS [120]. Wang et al. utilized a layer-by-layer methodology to fabricate a guanidine-functionalized (GF)-covalent organic framework (COF) nanocomposite, which was designed to capture EXOs and phosphopeptides. A substantial quantity of binding sites was available for AuNPs on Fe_3_O_4_@COF, which underwent amine group functionalization via polyethyleneimine (PEI). Guanidine was grafted onto the composite, which was subsequently designated Fe_3_O_4_@COF@Au@PEI-GF. The EXOs were isolated from a human serum sample, and TEM analysis revealed that their dimensions ranged from 30 to 150 nanometers. By monitoring the dynamic Brownian motion of the NPs in real time, NTA was utilized to estimate the EXO concentration. Approximately 1.2 × 10^9^ EXOs/mL were discovered to be present in the collected EXO. The phospholipid layer of EXOs and the guanidyl groups of Fe_3_O_4_@COF@Au@PEI-GF interact to explain the exosomal capture process [121]. Jang et al. investigated the method of protein-based detection. Transferrin-coated magnetic NPs (MTNs) were utilized to isolate EXOs derived from brain circulation in the context of neurological diseases. NPs of silica-coated Fe_3_O_4_ (Fe_3_O_4_@SiO_2_-NH2) were produced and subsequently conjugated to transferrin. In this instance, transferrin functioned as a ligand by binding to the transferrin receptor located on the EXO surface. The research investigated the potential of MTNs for various theranostic applications related to EXOs, in addition to their use in isolating EXOs derived from blood [122, 123].

Researchers, two coexpressed proteins, significantly enriched in CD24 and EpCAM, were found using bioinformatics analysis of ovarian cancer and EXO proteomes. Additionally, an as-derived dual-aptamer targeted EXO-based method for ovarian cancer screening was developed. To put it briefly, a rolling circle amplification chemiluminescent signal was created by a DNA ternary polymer that included aptamers targeting EpCAM and CD24. This polymer was engineered to exhibit a logic gate response upon identifying ovarian cancer EXOs. By measuring EXOs, a dynamic detection range of six orders of magnitude was attained. Furthermore, this approach may effectively distinguish EXOs from healthy individuals, other cancer patients, and people with ovarian cancer in clinical samples, allowing for potential in situ detection. The inadequate specificity of conventional protein markers was overcome by carefully choosing biomarkers and developing a dual-targeted exosomal protein detection approach. Through the discovery of EXO protein indicators unique to the illness, this study advanced the development of EXO-based prognostic monitoring in ovarian cancer [124].

## Optical biosensors for EXO detection

**Fig. 4:**
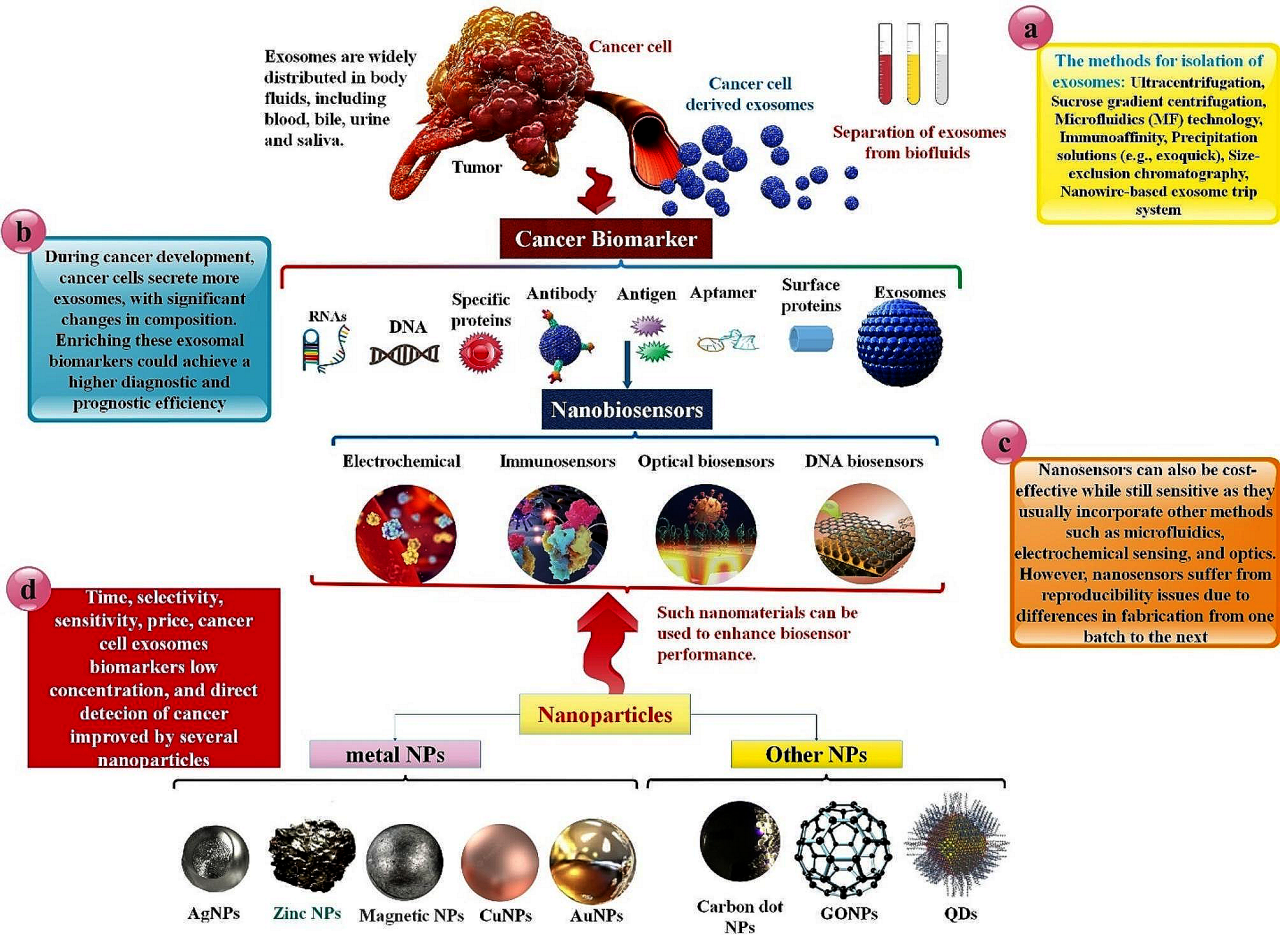
**a**,**b**) Schematic representations of various techniques for isolating and purifying extracellular material are provided. EXO purification techniques are both traditional and contemporary. For EXO separation, differential ultracentrifugation and size-exclusion chromatography are two of the most frequently employed techniques. To isolate specific varieties of vesicles, immunoaffinity absorption employs antibodies that target proteins present on the EXO surface. To capture EXOs efficiently, the microfluidics (MF) method utilizes circuits equipped with antibody-interceded connections. For ultrafiltration to generate a filtrate dense with vesicles possessing the optimal dimensions, a filter with a specific pore size is necessary [132]. **c**) EXO detection is possible via various platforms, including those based on electricity, DNA, immunosensors, electrochemistry, and optics. **d**) Nanomaterials are frequently employed as transducer materials, which play a critical role in advancing biosensors. A biosensor comprises the following four components: a bioreceptor, a transducer, a signal processor (which converts an electronic signal into the desired signal), and a display interface. Various metal NPs, including AgNP, AuNPs, and various GO, carbon dot, and QD NPs, are designed to facilitate specific procedures and serve as distinctive nanoplatforms for the detection of circulating biomarkers. The method that has been developed exhibits sensitivity, selectivity, and accuracy. Consequently, it presents a novel prospect for using EXO-based nanobiosensors in the early detection of cancer in human serum

Liquid biopsies have garnered significant attention in clinical medicine over the last several decades because of their intriguing potential for early diagnosis, illness evaluation, and tumor characterization [125]. Detection of EXOs, cell-free DNAs (cfDNAs), and circulating tumor cells (CTCs) is presently regarded as one of the most prominent areas of liquid biopsy analysis. However, adequate and dependable quantitative methods for detection are still lacking in this field. Significant technical advancements and the validation of novel concepts about diverse detection methods are imperative before advancing EXO-driven liquid biopsy toward its ultimate clinical application and disease monitoring. Hence, investigating detection modalities appropriate for high-throughput screening, real-time analysis, small sample volume, and LOD will be crucial in this domain [126]. Currently, optical techniques have shown exceptional stability and precision while assessing biological targets [21]. Optical biosensors accomplish their detection function by interacting optical fields with biorecognition components [127]. They are among the most widely utilized biosensors because of their tiny footprints, sensitivity, and specificity [128]. In general, optical biosensors fall into one of two categories: label-free or label-based. Label-free sensing denotes a method in which the signal is produced directly by the sample’s interaction with the transducer. The label-based approach requires the tagging of the target analyte with a reporter molecule to facilitate detection through the utilization of fluorescent, luminescent, or colorimetric signals [129]. Optical biosensors such as fluorescent, SPR, interferometer, optical waveguide, and ring resonator are often observed. When polarized light is illuminated at a certain angle, the SPR phenomenon occurs at the interface between the two conducting materials (such as glass and metal). A surface plasma wave will be produced as a result. Since the wave is located at the border between the conductor and the external medium (such as air, water, or vacuum), any modification to this barrier, such as molecules being absorbed by the conducting surface, would cause the oscillations to become very sensitive. Researchers can determine the detection result by tracking changes in wavelengths, reflectance, and angles over time [130]. Moreover, optical biosensors possess a low detection limit, high sensitivity, and are portable and real-time; they also have the potential to diagnose a wide variety of cancer types [131] (Fig. 4).

### Optical biosensors colorimetry

**Fig. 5:**
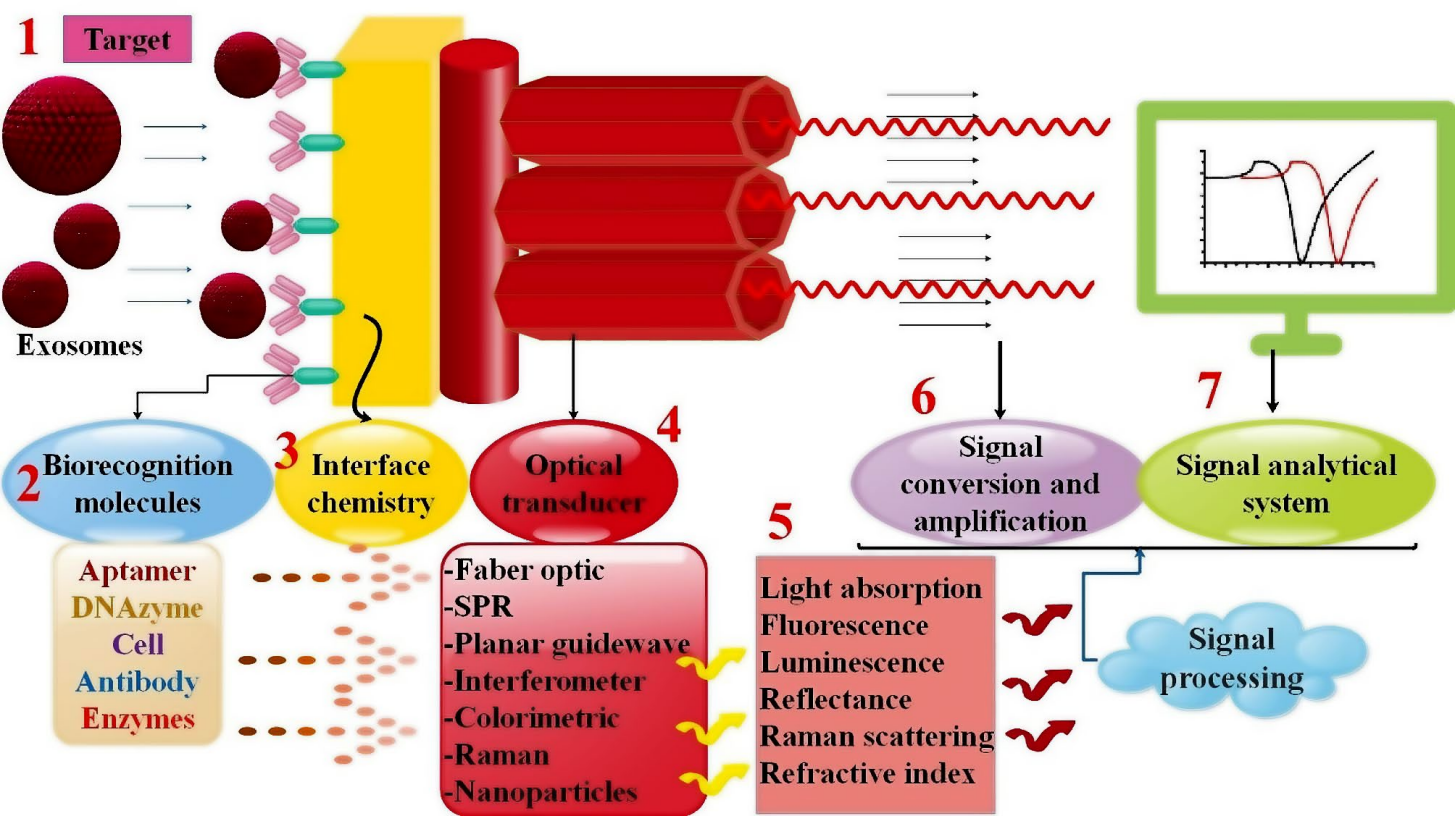
Figure of an optical biosensor. A compact analytical device that uses an optical transducer system and a sensing element for biorecognition is called an optical biosensor. Three methods that use the evanescent field near the biosensor surface to detect the analyte-biorecognition element interaction are optical waveguide interferometry, surface plasmon resonance (SPR), and evanescent wave fluorescence

One of the easiest methods for biosensing to employ is the colorimetric technique, which has the benefit of being simple to use. Colorimetric biosensors often display detection findings as discernible color changes that can be seen with the naked eye. UVeVis spectrophotometric measurement may be used to acquire quantitative data further [133]. Due to these qualities, colorimetric biosensors are a practicable and cost-effective method with vast potential for biomedical applications [134]. Aptasensors comprise most of the recently described colorimetric biosensors for EXO detection, most likely due to their particular adaptive binding characteristic [135]. Indicators comprised of AuNPs are widely used in colorimetric sensors. In their study, Jiang et al. presented a colorimetric biosensor that utilizes a panel of aptamers specific for EXO markers and AuNPs to enable EXO detection with the unaided eye within minutes [136]. Because of the particular interaction between the aptamer and exosomal protein, the noncovalent aptamer/AuNP complexations inhibit the aggregation of AuNPs and exhibit red when the complexation is disrupted in the presence of EXOs. The aptamer is removed from the AuNP surface because of this process, which also leads to the eventual aggregation of AuNPs and a reddish-blue color shift. This design exhibited the colorimetric profiling of several exosomal proteins and effectively recognized CD63 proteins from HeLa cell EXOs. Another aptamer/AuNP format colorimetric biosensor employs hydrogen peroxide and 3,3’,5,5’-tetramethylbenzidine as a chromogenic system [137]. The biosensor exhibits remarkable sensitivity in detecting HIF-1a (an early biomarker for myocardial infarction) from circulating EXOs in rat serum, with an LOD of 0.2 ng/L. This development introduces novel prospects for the early diagnosis of myocardial infarction. In a similar vein, Xia et al. investigated a colorimetric approach through the integration of aptamer and water-soluble single-walled CNTs (s-SWCNTs) to identify EXOs [138]. Numerous teams have used on-chip colorimetric detection to measure and describe EXOs. An EXO-specific multiplexed microfluidic platform with electrodes functionalized with antibodies was described by Vaidyanathan et al. The method is predicated on the surface shear force—also called nano shearing—caused by alternating current electrohydrodynamics (ac-EHD), which induces fluid movement within a few nanometers of the electrodes. EXOs were detected and quantified with excellent specificity using the colorimetric solution’s absorbance measurement. This platform outperformed existing fluid dynamic-based methods regarding sensitivity (2760 EXOs µL^− 1^) [139]. The successful implementation of a colorimetric aptasensor that is sensitive and selective has enabled the detection of EXOs derived from malignancy. The chromogenic signal was generated through the in situ deposition of polydopamine (PDA) and the polymerization of horseradish peroxidase (HRP)-accelerated dopamine (DA) [140]. A two-step sensing technique-based plasmonic colorimetric biosensor with exceptionally high sensitivity for EXO quantification was documented. The methodology utilized etching and a competitive reaction induced by EXOs to produce Au nanobipyramid@MnO_2_ nanosheet nanostructures. The signal of EXOs was converted to the quantity of ALP via a competitive reaction induced by EXOs; this facilitated the experimental procedure and enhanced the signal. Through ascorbic acid etching of the Au NBP@MnO_2_ NSs, the refractive index of gold nanobipyramid (Au NBPs) was increased. By leveraging the strengths of signal amplification and exceptional refraction, the LOD was established at 1.35 × 10^2^ particles µL^− 1^, which surpasses the sensitivity of colorimetric methods previously documented for EXO detection [141] (Fig. 5).

The study’s authors devised a trimode aptasensor that combines photothermal, fluorescence, and colorimetric capabilities with magnetic control to detect EXOs derived from human gastric cancer cells (SGC-7901). The functionality of this sensor was established upon CP/Mn-PBA DSNBs nanocomposites, which were synthesized through the deposition of copper peroxide (CP) nanodots onto double-shelled nano boxes modified with polyethyleneimine and manganese Prussian blue analogs. Apt-CP/Mn-PBA DSNBs, labeled with CD63 aptamers, were affixed to complementary DNA-labeled magnetic beads (cDNA-MB) via self-assembly. Aptamers formed preferential complexes with EXOs during incubation; the released CP/Mn-PBA DSNBs were effectively extracted by researchers via magnetic separation. Under near-infrared (NIR) light, the CP/Mn-PBA DSNBs demonstrated notable photoreactivity and photothermal conversion efficiency. Specifically, when exposed to 808 nm irradiation, temperature fluctuations occurred, which were correlated with varying concentrations of EXOs. In addition, colorimetric detection was accomplished by observing the color change in a 3,3′,5,5′-tetramethylbenzidine (TMB) system, which was made possible through the modification of PEI. The NIR-enhanced peroxidase-like activity of CP/Mn-PBA DSNBs and their ability to produce Cu^2+^ and H_2_O_2_ in acidic environments were also investigated. Furthermore, DNA sequences could generate dsDNA-templated CuNPs that emitted intense fluorescence at approximately 575 nm when Cu^2+^ and ascorbic acid (AA) were present. There was a positive correlation observed between EXO concentrations and reductions in temperature, absorbance, and fluorescence intensity. The trimode biosensor exhibited satisfactory capability in distinguishing human serum samples from those of healthy individuals and those of patients diagnosed with gastric cancer [142].

A dual-modal sensor is being developed by researchers, which integrates electrochemical assay with naked-eye detection of exosomal surface proteins originating from breast cancer. Most current sensors depend on aptamers that simultaneously recognize EXOs and generate amplified signals; therefore, precisely engineered aptamer probes are necessary to circumvent challenges associated with EXO identification. Aptamers unbound by EXOs can function as comprehensive templates for inducing the formation of G quadruplexes. Catalyzed substrates by the peroxidase activity of the G-quadruplex/hemin DNAzyme can produce both color and electrochemical signals. The dual-modal sensor that has been developed exhibits an exceptional ability to distinguish between healthy individuals, metastatic breast cancer patients, and nonmetastatic breast cancer patients by analyzing exosomal surface proteins. The sensor’s cost-effectiveness, simplicity, and universal applicability establish it as a potentially valuable diagnostic instrument in breast cancer research and clinical practice [143].

The two primary components of the sensing technique are the etching of Au nanobipyramid@MnO_2_ nanosheet nanostructures (Au NBP@MnO_2_ NSs) and the EXO-triggered competitive reaction. EXOs may transform their signal into the quantity of ALP via a competitive interaction with placeholder chains that they create. This simplifies the experiment and increases the signal. Ascorbic acid, which is produced when l-ascorbic acid 2-phosphate is hydrolyzed by ALP, etching Au NBP@MnO_2_ NSs and causing a blue shift in the longitudinal localized surface plasmon resonance peak, alters the refractive index of Au NBPs. With a detection limit of 1.35 × 10^2^ particles/µL ^–1^, this protocol shows high sensitivity toward EXOs within 8.5 × 10^2^ to 8.5 × 10^4^ particles/µL ^–1^, outperforming previously published colorimetric methods. This is due to the competitive reaction’s signal amplification and the superior refractive index sensitivity of colorimetric substrates. Furthermore, by varying the aspect ratio of Au NBPs, a sensitive multicolor visual detection of EXOs was achieved. It is important to note that the Au NBP@MnO_2_ NSs were created by growing MnO_2_ nanosheets in situ on Au NBPs. Because of their appealing optical characteristics and simplicity of etching, Au NBP@MnO_2_ NSs are intriguing options for plasmonic detection [144].

In an inquiry, scientists combined stimuli-responsive DNA microcapsules with acetylcholinesterase to generate acetic acid, and this resulted in the development of a pH-sensitive colorimetric biosensing technique for EXO identification. Acetylcholinesterase was placed into DNA shells that had been crosslinked using anti-CD63 aptamers to create the artificial DNA microcapsules. When EXOs were added, an energetically stabilized aptamer-CD63 complex was made, and the interaction between the aptamer of CD63 and the surface protein of EXOs caused the microcapsules to break apart, releasing the encapsulated AChE. Unreacted DNA microcapsules were removed using a straightforward centrifugation separation method, and the supernatant containing the released acetylcholinesterase was collected. This was used to detect colorimetric EXOs because the acetylcholinesterase hydrolyzed the acetylcholine to release acetic acid. A phenol red indicator was used to determine the ensuing lower pH of the solution; the abrupt color shift was quickly visible to the unaided eye. By using the solution’s absorption intensity ratio of 558 vs. 432 nm, EXO quantification was also accomplished. The limits of detection and quantification were 1.2 × 10^3^ and 2.2 × 10^3^ particles/µL, respectively, while the linear range was 2.0 × 10^3^ to 5.0 × 10^5^ particles/µL. Furthermore, this suggested method for detecting EXOs had a high recovery efficiency (> 94%) and a relative standard deviation of 3.1%, indicating a promising future for EXO research [145].

### Fluorescence nanotechnology methods for EXO detection

Fluorescence spectroscopy is based on the phenomenon wherein atoms emit light upon returning to their fundamental state after a prior excitation procedure. The development of fluorescence-based biosensors and optical bioassays extensively uses fluorescent probes [146]. Their transduction technique is predicated on the variations in wavelength or intensity of fluorescence that arise from the interaction of the fluorescent probes with the analyte. Following this productive interaction, the analyte may be precisely quantified by comparing variations in fluorescence to the starting concentration [116]. Although these methodologies exhibit exceptional analytical capabilities, their applicability is occasionally restricted due to compromised fluorophore stability. To surmount the limitations of existing fluorophores, fluorescent NPs possess favorable optical characteristics, including high quantum yields and photostability, low photobleaching, size-tunable emission, an extensive excitation range, and narrow emission that permits a significant Stokes shift [147].

Compared to traditional fluorescence detection approaches, these features allow for improved LOD and even single molecule detection, making fluorescence-based nanobiosensor devices more dependable and sensitive. In addition to silica and organically modified silica-based NPs, other fluorescent nanomaterials include metals, metal oxides, metal nanoclusters, organic polymers, quantum dots (QDs), silicon QDs, and various carbonaceous nanomaterials like carbon dots, CNTs, carbon nanoclusters, and nanodiamonds [148–150]. UCNPs doped with lanthanide experience a non-linear photophysical transformation in which low-energy radiation, predominantly in the NIR spectrum, is transformed into higher-energy radiation, such as visible light (anti-Stokes shift). Therefore, lesser wavelengths correspond to the emission of fluorescence from UCNPs as opposed to light absorption. This characteristic renders them particularly appealing for biological applications and the advancement of nanobiosensors, as it circumvents the need for ultraviolet (UV) light, thereby reducing the autofluorescence exhibited by biological samples [151, 152]. In creating aptasensors based on Luminescence Resonance Energy Transfer (LRET), UCNPs linked to aptamers have been employed as energy donors in conjunction with other fluorophores or NPs that function as acceptors. When the energy levels of two luminescent or fluorescent molecules interact over very short distances—that is, when the excitation wavelength of the acceptor molecule and the emission wavelength of the donor molecule coincide—LRET and FRET are the mechanisms that result. This is how the excited donor gives energy to the acceptor, causing it to release a photon. Tetramethyl rhodamine (TAMRA) and uncoupling polymer NPs (UCNPs) functionalized with two DNA aptamers were used by Wang et al. to target the epithelial cell adhesion molecule (EpCAM) found on the EXO membrane of specific cell types [153]. The LRET process was facilitated by the proximity of both DNA strands and the reduction in distance between the energy donor (UCNPs) and the acceptor (TAMRA) after the aptamer-EXO binding. By the correspondence between the emission wavelength of the donor and the excitation spectrum of the acceptor, UCNPs excitation by IR light generates a UV emission that stimulates the TAMRA molecule, resulting in an EXO concentration-dependent yellow emission (585 nm). The LOD attained by this LRET sensor was 8 × 10^4^ particles per mL [154]. By using variations in fluorescence signals, fluorescence may be utilized for qualitative and quantitative analysis. Fluorescence technologies are user-friendly, offer excellent selectivity and sensitivity, and are very sensitive. The concentration of EXOs has been measured using a variety of nanomaterial-based fluorescence biosensors that combine fluorescence technology with nanomaterials donors of resonance energy. To detect EXOs extracted via the ultracentrifugation technique, Chen et al. developed a paper-based aptamer sensor based on resonant energy transmission between upconversion NPs and AuNPs [155]. Two segments of the aptamer could bind to the CD63 protein on the EXOs’ surface in the presence of EXOs; this would reduce the distance between the upconversion NPs and the Au NPs, thereby facilitating luminescence quenching. The fluctuations could be observed through the utilization of a do-it-yourself imaging system. To quantify the luminescence, the intensity of the green channel in the resulting color image was extracted by Photoshop software. The relationship between the concentration of EXOs and the quenching of upconversion nanoparticle luminescence (1.0 × 10^7^–1.0 × 10^11^ particles/mL) was linear, suggesting that EXOs could be detected. By utilizing up-conversion NPs as a luminescent material and testing a 10-M sample of EXOs extracted from human liver cancer HepG2 cells in PBS (1.1 × 10^6^ particles/mL), this method successfully achieved the low detection limit. Following this, they devised a washing-free aptasensor for detecting EXOs, which was constructed using tetramethyl rhodamine acceptors and rare-earth-doped upconversion nanoparticle donors. A reduction was made in the LOD to 8.0 × 10^4^ particles/mL [153, 156, 157].

Since their inception, fluorescent molecules have been among the most widely used reporters and transducers in biosensor construction. This is attributable to their exceptional sensitivity, speedy response, and adaptability. As of this moment, fluorescent biosensors have been extensively implemented on a vast array of targets, including EXOs. EXO detection using a label-free and activatable fluorescent DNA aptasensing method based on the strand replacement reaction and the formation of G-quadruplex was described by Chen et al. [158]. The development of a homogeneous magneto-fluorescent EXO (hMFEX) nanosensor enables the rapid analysis of EXOs derived from tumors [159]. Using this platform, tumor-derived EXOs could be identified with exceptional sensitivity and specificity. With a LOD of 6.56 × 10^4^ particles µL^− 1^, this method exhibits promise as a clinical diagnostic tool. It has been demonstrated that EXO assays based on fluorescent silicon NPs (SiNPs@EXO) can effectively quantify and distinguish between EXOs derived from normal and metastatic sentinel lymph nodes (SLNs) [160]. To investigate the EXOs produced by the human embryonic kidney (HEK293T) and mouse breast cancer (4T1) cells, 1,8-napthalimide fluorophores were utilized. The fluorescence intensity of EXOs captured by 4T1 cells was more significant than the negative control (HEK293T). The fluorescence analysis revealed that the signals in metastatic SLNs peaked within thirty minutes and remained there for up to 3 h. In contrast, the signals in normal SLNs peaked within an hour and then decreased abruptly. By utilizing this integrated methodology, lymphatic metastasis can be predicted by identifying EXOs derived from SLN [141].

Researchers have devised a one-step, label-free technique for detecting plasma EXOs using ratiometric fluorescence. In particular, bicolor streptavidin magnetic beads were made with a green color SYBR Green I (SGI) incorporated in the stem of Cy5-Apt to react to EXOs and an immobilized Cy5-labeled hairpin aptamer for CD63 (Cy5-Apt) on its surface to detect EXOs. EXO measurement based on the fluorescence ratio of Cy5 and SGI is made possible by Cy5-Apt’s potential conformational change during EXO capture, which releases the contained SGI. The suggested approach offers a substantial advance over the prior method in that it can accomplish the enrichment, separation, and detection of EXOs in a single step (30 min). Additionally, EXO enrichment and interference removal from complicated matrices are made possible by the use of ratiometric fluorescence and magnetic separation, which increases sensitivity and accuracy. With an area under the receiver operator characteristic curve of 0.85, the test in particular can differentiate lung cancer patients from healthy volunteers and identify EXOs in plasma. Additionally, by just altering the aptamer, researchers were able to create a practical approach for assessing the different divisions of EXOs, which has significant potential for point-of-care applications [161].

An additional investigation produced innovative lateral flow test strips utilizing aptamer-functionalized fluorescent microspheres (FMs-aptamer) and MnO_2_ to enable colorimetry/fluorescence dual-mode sensing of cancer-associated EXOs in serum samples (using MCF-7 cell-derived EXOs as a model). A dual-mode output platform was fabricated by effectively utilizing the size advantage of EXOs to mediate the fluorescence resonance energy transfer (FRET) between FMs (donors) and MnO_2_ (acceptor). By measuring the variation of two distinct signals, researchers proposed a method that could reduce the occurrence of false positives compared to single-readout test strips. The test strip effectively and precisely detected MCF-7 EXOs, with a LOD of 2.5 × 10^3^ particles/mL. The platform’s capability to promptly and directly identify cancer EXOs in serum samples, without laborious sample pretreatment, demonstrates its significant potential as a tool for point-of-care testing [162].

### Surface plasmon resonance (SPR) detection

SPR is a phenomenon in which particles of incident light with a particular angle of incidence excite electrons in a metal surface layer, which then propagate in a parallel direction with the metal surface. The specific angle at which SPR is initiated with a thin metal surface and a constant light source wavelength is determined by the material’s refractive index near the metal surface. Consequently, even a minor alteration in the reflective index of the sensing medium will impede the occurrence of SPR, thereby enabling the detection of analytes. SPR assays are label-free and real-time detection methods because the concentrations of analytes are calculated by tracing the resonance angle variations or monitoring the intensity of the reflected light. To date, many SPR biosensor variants have been created to identify clinically significant biomarkers. Additionally, numerous nanomaterials are used to enhance the efficacy of SPR biosensors [163]. In conventional SPR, thin films of metals such as Au, Ag, or aluminum can be used to determine the molecular concentration for biosensing. The angle of incidence at which the minimal light intensity is reflected between the metal and dielectric boundary is referred to as the SPR angle. There exist various methodologies for augmenting the performance of biosensors. Abdulhalim et al. enhanced the biosensor’s functionality by integrating a metallic sheet with a high refractive index [164]. For example, the SPR of AuNPs, the emission wavelength of CNTs or QDs, and the magnetic properties of magnetic NPs (MNPs) are significant attributes that can be customized as needed. A widespread benefit of NPs is their substantial surface area to volume ratio, facilitating the adsorption of a greater quantity of biomolecules [165].

To analyze molecular interactions, SPR is a physical optical phenomenon resulting from the complete reflection of light at the interface of a metal film and a liquid. A nano-plasmonic EXO (nPLEX) assay was devised by Im et al., utilizing periodic nanohole arrays and transmission SPR. Functionalized antibodies in each array caused spectral shifts or variations in intensity on the nPLEX sensor, which were directly proportional to the concentrations of target exosomal proteins [166]. A distinct enhancement in the intensity and wavelength shift of scattering was observed on AuNC-EXO-AuR in an alternative investigation attributed to the plasmon effect [167]. Using anti-LRG1 antibody-conjugated AuR probes, the differential expression of LRG1 (leucine-rich alpha-2-glycoprotein 1) in urinary EXOs of lung cancer patients and healthy individuals was analyzed. An additional biosensing assay utilizing SPR imaging (SPRi) was invented by Fan et al. A modified Au chip containing antibodies (anti-CD63/anti-EGFR/anti-EpCAM) and various recognition sites enabled the multiplex characterization of EXOs derived from NSCLC via bio-affinity interactions. It was estimated that the LOD for this biosensor was 10^4^ particles/µL. Despite their numerous advantages, the challenging fabrication process of nanostructures restricts the widespread implementation of nanoplasmonic biosensors. To tackle this obstacle, Liu et al. devised a nanostructure-free intensity-modulated SPR biosensor. The reflection and reference intensities of lasers were measured by two photodetectors in this sensor, which was employed to quantify the levels of exosomal protein expression. Notably, this sensor demonstrated a greater sensitivity to detection than ELISA [168].

Optical apparatus designed to detect biological analytes with exceptional sensitivity via SERS spectroscopy. SERS is a susceptible optical detection method that employs lasers to stimulate vibrational transitions in molecules that are adsorbed onto the surface of metal NPs [169]. SERS has become recognized as an up-and-coming platform for many biosensing applications. sensing as mentioned earlier systems incorporate the benefits of high specificity, exceptional sensitivity, stability, affordability, repeatability, and user-friendly procedures. Furthermore, the capability of SERS to provide a molecular fingerprint and detect the target analyte even at low concentrations renders it an auspicious method for the more precise and dependable detection of circulating cancer biomarkers. On the list of circulating biomolecules, oncomiRs are gaining recognition as significant biomarkers in the context of breast cancer (BC) early detection [170]. A novel assay for the protein profiling and real-time detection of EXOs was documented. To capture EXOs, Au-plated slides were typically utilized in conjunction with 3D-printed antibody arrays. For the quantitative detection of target proteins, QSY21-coated Au nanorods were employed as the label agent. The quantification of plasma-derived EXO levels in patients diagnosed with breast cancer was achieved through the utilization of an assay that targeted HER2 and EpCAM. The suggested 3D-printed array template facilitated the establishment of a detection platform that was inexpensive, portable, and readily accessible; this introduced a novel approach to the advancement of liquid biopsy for cancer [168]. Dong et al. demonstrated that evaluating protein phosphorylation status could offer novel prospects for the detection of cancer [171]. An unlabeled macroporous inverse opal structure resembling a beehive, coated in Au, was devised to capture and analyze EXOs. The intensity of the 1087 cm^− 1^ SERS peak, which corresponds to the P-O bond present in the phosphoproteins of EXOs, was significantly higher in the plasma of cancer patients than healthy donors, by at least twofold. Nevertheless, the two assays as mentioned earlier necessitate the pre-separation of EXOs, thereby significantly impeding the speed of analysis. Pang et al. introduced a straightforward immunoassay for the direct acquisition and analysis of exosomal PD-L1 from serum samples [172]. To isolate EXOs, Fe_3_O_4_@TiO_2_ NPs were utilized, while anti-PD-L1 antibody-modified Au@Ag@MBA SERS tags were implemented to label EXOs with PD-L1 and enable SERS detection. The ability of this assay to quantify exosomal PD-L1 in a 4 µL serum sample within 40 min was validated [24] (Table 1).

In a separate study, an effective technique for label-free EXO profiling in biological samples (such as plasma) has been proposed. It combines deep learning with surface-enhanced Raman spectroscopy (SERS) on MXene-coated gold@silver core@shell NP (Au@Ag NP) functionalized substrate. The as-proposed SERS sensing platform exhibits a dynamic range of 0.5 × 10^10^ to 2.0 × 10^11^ EVs mL^− 1^, with a LOD as low as 1.7 × 10^9^ EVs mL − 1 (three times the standard deviation (3σ) of blank sample. This is attributed to electromagnetic enhancement (EM) and chemical enhancement (CM) of MXene-coated Au@Ag NP substrate. The remaining neural networks are then used to extract the characteristics of EVs from complicated Raman spectra using a deep-learning classification approach. As a proof of concept, the first validation of the researchers’ method is shown by the 96.0% diagnostic accuracy in separating thyroid cancer patients from healthy controls and the 86.6% accuracy in staging the cancer patients, respectively [173].

A substance that targets human HER2, trastuzumab is an efficacious treatment for breast cancer with metastases. Nevertheless, certain patients may resist this treatment; thus, it is critical to monitor its efficacy. In this article, researchers describe deep learning-assisted monitoring of trastuzumab efficacy using urinary EXOs from mice overexpressing HER2. For the SERS immunoassay, individual Raman reporters containing the intended SERS tag and EXO capture substrate were fabricated. The signals emitted by the SERS tags were gathered to generate training data for deep learning. The deep learning algorithm effectively quantified and classified various complex SERS tag mixtures. Comparative analysis was conducted between the exosomal antigen levels determined by SERS-deep learning and those obtained by quantitative reverse transcription polymerase chain reaction and western blot for five distinct types of cell-derived EXOs. Finally, drug efficacy was evaluated using urinary EXOs extracted from trastuzumab-treated rodents and SERS-deep learning analysis. By utilizing this monitoring system, proactive responses to treatment-resistant issues should be possible [174].

Only by creating very effective EXO separation methods can significant and influential advances in SERS-based EXO research be possible. In addition to improving the quality of separated EXOs, careful selection of isolation techniques suited to particular biological samples and cargo types being tested will guarantee the validity of the SERS findings. One effective way to expedite the separation and analysis procedure is to combine label-free SERS analysis automation with microfluidic devices. Label-free SERS-based EXO research may become much more reliable and efficient overall with this integration, which may also lower sample volume needs. Moreover, creating magnet SERS substrates offers a fascinating method that combines EXO detection and separation. This new method has great potential for high-throughput SERS analysis and presents fascinating opportunities for further EXO study. SERS-based label-free EXO detection has promise, but to adequately support clinical and research applications, several important issues need to be resolved. EXOs constitute a significant challenge for SERS-based detection due to their fantastic variation regarding size, content, and surface proteins. The development of adaptable SERS substrates and analytical techniques that can handle this variability is required to meet this issue. Moreover, even though SERS is known for its remarkable sensitivity, it is still difficult to reliably obtain low EXO detection limits. Low concentrations of EXOs are challenging to detect due to their intrinsically low refractive index and feeble Raman scattering signal. As a result, it is urgent to investigate novel SERS substrates with enhanced sensitivity and to develop sophisticated signal amplification techniques that can further the frontiers of EXO detection. Therefore, researchers thoroughly examine the substantial progress achieved in SERS substrates, acknowledging their critical significance in augmenting SERS analysis. The primary objective has been to surmount obstacles of reproducibility, signal uniformity, stability, and biocompatibility; this has resulted in the creation of many inventive approaches. Considerable investigation has been dedicated to examining the form, dimensions, and core-shell configuration of NPs to enhance the reliability and effectiveness of SERS signals. Furthermore, scientists have investigated a wide range of SERS substrate materials, such as porous materials, bacterial substrates, Pt-black SERS templates, and ITO glass, all of which provide distinct characteristics that contribute to improved performance. Furthermore, to enhance the quality of SERS signals, molecular linkers including ATPES and antibodies have been created and implemented. Molecular linkers are of utmost importance in enabling effective signal transduction via SERS and augmenting the analysis’s sensitivity and specificity. In response to this challenge, it is imperative to adopt proactive strategies toward creating economical SERS platforms and user-friendly devices that are easily implementable across a wide range of healthcare environments [175].

**Table 1:** Optical biosensors for detection exosomes

Optical biosensors	Sensitivity (LOD)	Explain	Ref
On-chip colorimetric detection	2760 EXOs µL^− 1^	The method is predicated on the surface shear force— called nano shearing—caused by alternating current electrohydrodynamics (ac-EHD), which induces fluid movement within a few nanometers of the electrodes.	[139]
Colorimetric aptasensor	7.77 × 10^3^ particle/mL	In this design, target exosomes (EXOs) were firstly captured by latex beads via aldimine condensation, followed by bio-recognition using a specific CD63 aptamer, which was conjugated to horseradish peroxidase (HRP) through biotin-streptavidin binding. Overall, a sensitive and selective colorimetric aptasensor was successfully developed for detecting cancer-derived EXOs facilitated by HRP-accelerated DA polymerization and in situ PDA deposition.	[140]
Plasmonic Colorimetric Biosensor	1.35 × 10^2^ particles µL^–1^	The sensing strategy mainly includes two steps: EXO-triggered competitive reaction and etching of gold nanobipyramid@MnO_2_ nanosheet nanostructures (Au NBP@MnO_2_ NSs). A competitive response between EXOs and placeholder chains induced by EXOs can translate the signal of EXOs into the amount of alkaline phosphatase, which simplifies the experimental process and amplifies the signal.	[144]
An aptasensor based on upconversion nanoparticles as LRET	80 particles/µL	Researchers proposed a washing-free aptasensor based on luminescence resonance energy transfer (LRET) between rare-earth-doped upconversion nanoparticles (UCNPs) donor and tetramethyl rhodamine (TAMRA) acceptor for susceptible detection of EXOs.	[153]
A paper-supported aptasensor based on upconversion luminescence resonance energy transfer	1.1 × 10^3^ particles/µL	Researchers used a simple paper-supported aptasensor based on LRET from UCNPs to gold nanorods (Au NRs) for the accessible determination of EXOs.	[155]

## Nanomaterials-based optical (nano) biosensors for the detection of EXOs in cancer

NP-based biosensors have emerged as a prominent subject within the diagnostics domain [176]. Utilizing NPs permits the creation of devices with enhanced sensitivity and LODs, characteristics that are gaining prominence in the sensing industry. In addition, lab-on-a-chip assays enable the rapid analysis of minute sample volumes, thereby decreasing the expense of clinical care. Numerous detection platforms for EVs have been documented, employing various sensing techniques, including colorimetry, fluorescence, SPR, SERS, electrochemistry, and nuclear magnetic resonance. These developments underscore the diagnostic potential of NPs. The advantages and disadvantages of these techniques will be elaborated upon in the following Sect. [154]. In particular, nanomaterials have a substantial effect on the domain of biosensors as they pertain to illness detection. When developing a biosensor for detecting cancer cells, the ability to detect deficient concentrations of biomarkers (and sometimes even a few cells) is one of the most critical factors. Despite their considerable contributions to cancer detection and diagnosis, conventional imaging techniques and procedures have limitations (e.g., time-consuming, imprecise, false-positive results) [177]. Nanomaterials have assumed the challenges and paved the way for resolving this issue. Significant characteristics, including chemical stability, electrical conductivity, a high surface-to-volume ratio, and adaptability to specific needs, have established nanomaterials as the most viable alternatives [178]. Determining the suitability of materials by the nature and function of sensors is a critical element in their design. To date, numerous types of nanomaterials, including metals, carbon nanomaterials, and metal derivatives, have been created. These materials have demonstrated commendable performance. Based on requirements, scientists have attempted to synthesize novel materials, thereby continuously expanding the range of possibilities [179].

Electrochemical biosensors make extensive use of metal NPs, including Ag and Au NPs, because of their exceptional stability and electrochemical, electronic, and catalytic properties [180]. Additionally, their surfaces permit simple functionalization. Substantial effort has been dedicated to developing electrochemical biosensors utilizing metal NPs. Quantitative EXO analysis was implemented on a microfabricated multichannel Au device developed by Zhou et al. The quantification of prostate-specific membrane antigen (PSMA) and epithelial cell adhesion molecule (EPCAM) expression was achieved through the oxidation of copper NPs and AgNPs, respectively [181]. When 25 µL of EXOs extracted from PCa clinical samples in phosphate-buffered saline (PBS) were utilized, a LOD of 50 particles/mL was achieved. The surface expression of EXOs from individuals diagnosed with prostate cancer exhibited a substantially higher level of PSMA and EPCAM compared to EXOs from healthy individuals. With the capability to monitor prostate cancer, this electrochemical sensor offers an effective substrate for protein marker detection from tumor-derived EXOs and microsomes [157].

Electrochemical and optical biosensors rely heavily on precious metal materials, including Au, Ag, platinum, and iridium, due to their exceptional biocompatibility [182], as well as their distinctive photoelectric properties [183]. Concerning optical sensors, ongoing development involves the creation of a sensor that exploits the variation in color intensity caused by the configuration of metal NPs [184]. Stability is increased in the domain of fluorescence by combining metal NPs with fluorescent probes; the dimensions of the metal NPs facilitate enhanced binding to ligands [185]. This could potentially improve the sensor’s response time. The sensor applications that incorporate these NPs of precious metals aim to increase the sensor’s sensitivity; the size and shape of identical precious metal particles impart distinct properties to the particles. This might be a viable option for additional enhancement [29].

### EXOs detection by using graphene oxide -modified optical biosensors in cancer

Graphene and GO are particularly well-studied materials within the biosensors industry. On its surface, GO contains oxygen-rich functional groups. Because it forms covalent bonds and is readily oxidized and acidified, it is highly amenable to chemical modification. In addition, studies have begun to utilize GO for probes, biological reagent analysis, and biological imaging in recent years, demonstrating its tremendous potential in the biomedical field on account of its favorable physicochemical and hydrophilic properties. Furthermore, GO exhibits advantageous properties such as high surface area, water solubility, and biocompatibility owing to its abundant functional groups, which enable it to be modified into biological probes. Previous research has demonstrated that GO can be efficiently utilized as a probe substrate to ensnare numerous small biological molecules, including DNA, bacteria, and cells [186]. GO and 5-carboxyfluorescein (FAM)-labeled DNA nano-biosensor was designed to identify the deletion mutation in exon 19 of the EGFR gene, which was responsible for the development of lung cancer. The approach relies on the adsorption of single-stranded DNA (fDNA) labeled with FAM onto GO, which subsequently inhibits the fluorescence of FAM. The formation of double-stranded DNA (dsDNA) from its complementary DNA (cDNA) led to fluorescence recovery from FAM through desorption and release from GO. Conversely, discordant DNA did not result in the formation of corresponding dsDNA; consequently, the fluorescence of FAM could not be restored [187].

Clinical diagnosis is aided by the analysis of cancer-related biomarkers, including circulating tumor cells (CTCs), circulating tumor DNA (ctDNA), microRNA, and EXOs, which provide vital information for the detection of tumor metastasis, postoperative recurrence surveillance, and early cancer diagnosis. Nevertheless, the clinical detection and analysis of specific tumor markers—including CTCs, ctDNA, and microRNA—are constrained by the low concentrations present in the blood. Due to their favorable physicochemical characteristics, GO-based nanomaterials are currently found in numerous biomedical detection technologies. Good hydrophilicity, mechanical flexibility, electrical conductivity, biocompatibility, and optical performance are characteristics of these materials. Furthermore, the implementation of GO as a biosensor interface has demonstrated a significant enhancement in the sensitivity and specificity of cancer detection biosensors [186].

An additional investigation reveals that quantitatively identifying CDE present in biofluids at low concentrations remains a formidable obstacle. This study presents the development of an electrically powered and label-free approach for directly detecting EXOs. The method is predicated on utilizing a reduced graphene oxide (RGO) field effect transistor (FET) biosensor. The fabrication of an RGO FET biosensor incorporating a specific antibody CD63 in the sensing region enabled the label-free and electrical quantification of EXOs. The method attained a detection limit of 33 particles/µL, considerably lower than the limits of numerous other methods presently accessible. Furthermore, the utilization of the FET biosensor to identify EXOs in clinical serum samples revealed substantial disparities between detecting healthy individuals and patients diagnosed with prostate cancer (PCa). This study presents an innovative technology that enables unlabeled direct quantification of EXOs, distinguishing it from other technologies and suggesting its potential as a tool for early cancer detection [76].

EXOs have demonstrated considerable promise as cancer biomarkers, and numerous cell-specific molecules have been identified within EXOs associated with colorectal cancer (CRC). In this study, scientists devised a DNase I enzyme-assisted fluorescence amplification technique to detect CRC EXOs via interactions between GO and DNA aptamers (CD63 and EpCAM aptamers). GO-quenched fluorophore-labeled aptamers regained their fluorescence upon incubation with samples containing CRC EXOs. As a result of the digestion of single-stranded DNA aptamers on the EXO surface by the DNase I enzyme, the EXOs were capable of interacting with a more significant number of fluorescent aptamer probes, which led to an augmentation in signal amplification. 2.1 × 10^4^ particles/µl is the LOD for CRC EXOs. As a result, a method characterized by its high sensitivity and rapidity was developed. Verified in 19 clinical blood serum samples, the technique distinguishes between healthy and CRC patients, demonstrating substantial diagnostic power. Furthermore, it is extensible to other types of EXOs besides CRC [188].

### Quantum dots-modified optical biosensors for detection of EXOs in cancer

QDs are distinguished from other fluorescent NPs by their exceptional electronic and optical characteristics. When comparing various forms of optical labels based on NPs, QDs exhibit notable benefits in the context of EXO detection. The diameter of QDs ranges from 2 to 10 nm, enabling them to label and detect EXOs within this smaller size range effectively. The fluorescence properties and photostability of QDs are exceptional. To detect EXOs with greater sensitivity, Hyung-Mo Kim et al. utilized multi-QDs encapsulated in silica-encapsulated NPs (M–QD–SNs), characterized by their brighter nature and consistent size compared to single QDs, in conjunction with the lateral flow immunoassay technique. EXOs from human foreskin fibroblasts (HFF) were detected using a lateral flow immunoassay employing M–QD–SNs to which anti-CD63 antibodies were secreted. The concentrations of EXO samples varied considerably, spanning from 100 to 1000 EXOs/µL. Investigators recently developed a system that achieved a detection limit of 117.94 EXO/µL, an eleven-fold reduction compared to the limits previously documented. Furthermore, EXOs were identified selectively compared to liposomes, neonatal calf serum, and negative controls, confirming that this technique effectively eliminated non-specific binding. Researchers’ study proves that lateral immunochromatographic analysis utilizing M–QD–SNs can efficiently and precisely detect EXOs with high sensitivity and quantitation [189, 190].

Based on CD81 protein expression, EXOs were captured by magnetic particles in this investigation. Researchers present an efficacious and straightforward approach to EXO detection in this investigation. They achieve this by employing QDs in combination with magnetic bead (MB) EXO isolation that is magnetic and targets tetraspanin CD81 EXO markers. It has been demonstrated that CD81 is a dependable marker for EXO identification. Low CD81 expression is observed on platelet cells, which are a significant source of normal EXOs in plasma. Thus, the contamination of normal EXOs is drastically reduced when CD81 capture ligands are utilized, which increases the sensitivity of EXO detection from tumor sources. While CD81 expression is not ubiquitous in all EXOs, it is frequently observed in various cancer EXOs. For EXO capture, scientists employ MB conjugated with CD81 antibody to circumvent the necessity for protracted sample purification. Investigators used universally available and highly fluorescent QD655 coupled with secondary antibodies for detection to identify primary antibodies that bind to surface protein markers of interest on EXOs. Various surface markers on EXOs derived from cells were utilized to evaluate this method in a breast cancer model. EXO HER2 has a high diagnostic capacity for HER2-positive breast cancer, as demonstrated by the method in this study. For fundamental vesicle research and clinical applications, researchers’ QD-based method with magnetic separation exhibits considerable potential in detecting EXOs at the molecular level. This is owing to its simplicity, speed, and low sample consumption. Protein markers of interest on the surface were identified using primary antibody-conjugated QDs followed by secondary antibody-conjugated fluorescent spectroscopy. Approved by ELISA, investigators’ methods can distinguish cancer EXOs from normal EXOs by specifically detecting various surface markers on EXOs derived from distinct cancer cell lines. Pilot plasma samples were utilized to illustrate the clinical potential, with HER2-positive breast cancer serving as the disease model. The findings indicate that the level of HER2 expression in EXOs obtained from patients diagnosed with HER2-positive breast cancer was five times greater than that of healthy controls. The diagnostic capability of exosomal HER2 for HER2-positive patients was substantial, as evidenced by its area under the curve of 0.969. This quantum dot-based EXO method is practicable for routine use in basic vesicle research and clinical applications, as it is rapid (less than 5 h) and requires only microliters of diluted plasma without pre-purification. In summary, the researchers have shown that exosomal surface markers can be detected quantitatively by combining QDs with magnetic separation with microbeads. The procedure involved the capture and concentration of EXOs onto magnetic microbeads. Subsequently, the QDs conjugated with secondary antibodies recognized the targeted surface markers. Researchers can precisely and quantitatively identify various surface protein markers present on EXOs derived from distinct breast cancer cell lines by employing this QD-based methodology. Cancer-associated surface protein markers may also be utilized by researchers to distinguish cancer EXOs from healthy EXOs. Using pilot clinical samples, researchers have demonstrated that HER2-positive breast cancer can be identified using QDs in conjunction with magnetic separation and enrichment to analyze HER2 expression on plasma EXOs. HER2 expression is approximately five times greater in cancer patients than healthy donors. Based on the AUC value of 0.96875, exosomal HER2 appears to be a highly effective diagnostic marker for patients who are HER2-positive [191].

**Fig. 6:**
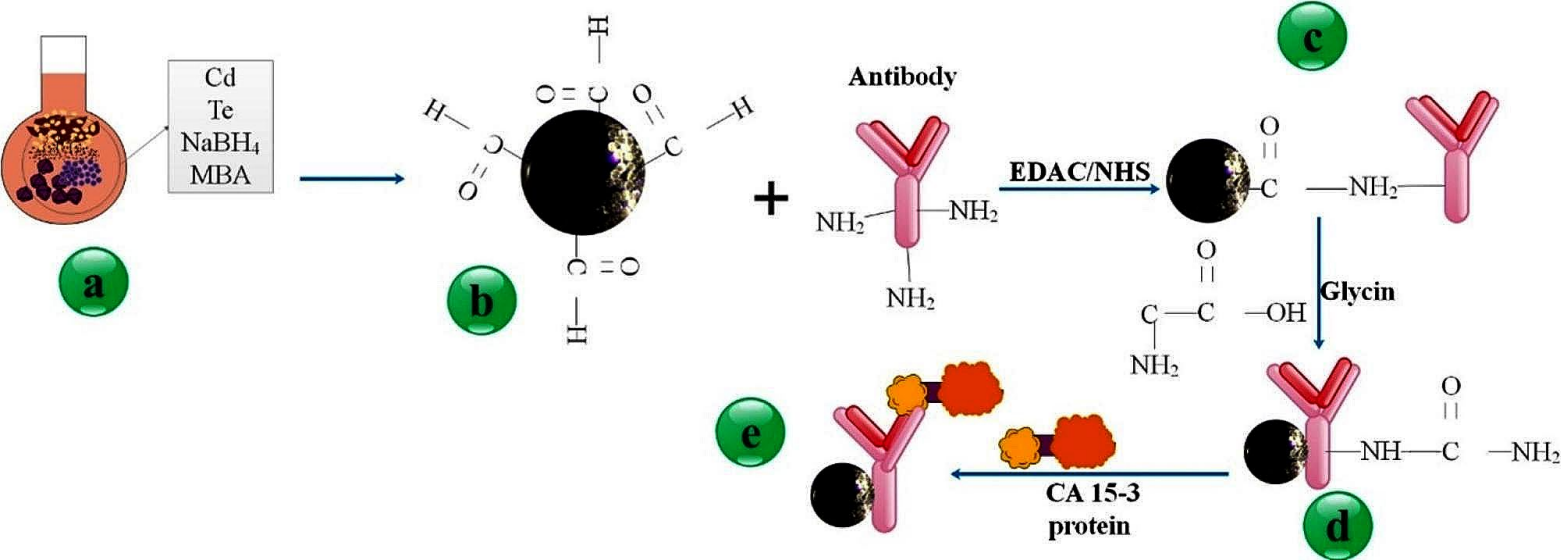
The schematic illustrates how the QD NPs were synthesized, and the QD conjugates were prepared. (A) Synthesis of QD; (B) Activation of carboxylic groups derived from QD; (C) Formation of a covalent bond via the EDAC/NHS reaction between the carboxylic groups of the NPs and the amine groups of the antibodies; (D) Obstruction of unbound regions. (E) QD conjugate binding to the CA 15 − 3 protein [192]

Cancer antigen 15 − 3 (CA 15 − 3) is a biomarker utilized in an additional study to monitor disease recurrence and treatment response in breast cancer. This study presents the fabrication and utilization of a fluorescent biosensor that utilizes mercaptopropionic acid-functionalized cadmium telluride (CdTe@MPA) QDs conjugated with CA 15 − 3 antibodies to detect the cancer antigen CA 15 − 3 protein tumor marker with ultrasensitivity. Initially, the QDs were produced through the hydrothermal method, yielding spherical NPs with a maximum diameter of 3.50 nm. The QD conjugates were subsequently characterized via fluorescence, Fourier transform infrared spectroscopy (FTIR), and UV absorption. Using fluorescence spectroscopy, the interaction between the conjugates and the protein was investigated in buffer and in commercial human serum that had been diluted by a factor of 10. The detection limit of 0.027 U/mL obtained through calibration in contaminated serum samples is one thousand times lower than the clinical limit for CA 15 − 3 (25 U/mL to 30 U/mL), proving this method is ultrasensitive. Furthermore, a prompt response was achieved in ten minutes. In the presence of the interfering serum proteins BSA, CEA, and CA-125, the biosensor exhibited selectivity, with BSA causing a maximal interference of 2%. The recovery percentage approached 100%, as indicated by the maximal relative standard deviation (RSD%) of 1.56%. In general, the CA 15 − 3 biosensor that was developed offers a straightforward and vigilant approach to ultrasensitive breast cancer surveillance, in addition to its capability of identifying other target molecules in human serum matrices. The extensive investigation of fluorescent biosensing devices for point-of-care applications is attributable to their affordable price, rapid and straightforward reading, and high sensitivity. A novel QD-based biosensor for detecting and quantifying the CA 15 − 3 protein in human serum samples was developed in this study. The detection was accomplished through the direct conjugation of CA 15 − 3 antibody with red-emitting QD NPs. The biosensor demonstrated a linear relationship between the fluorescence intensity of CA 15 − 3 and its concentration in the sample and buffer, as well as in human serum. In 10-fold diluted human serum, the LOD was 0.027 U/mL. Moreover, when applied to loaded human serum samples, the biosensor demonstrated high recovery ratios, indicating that it is a cost-effective and straightforward method for quantifying the analytes of interest (Fig. 6) [192].

#### Graphene quantum dots-based optical biosensors for detection of EXOs in cancer

Graphene quantum dots (GQDs), a novel category of fluorescent materials belonging to the carbon nanomaterials family, exhibit optimal chemical and physical characteristics that render them suitable for integration into sensors designed for medical and biological purposes [193]. From a morphological standpoint, GQDs exhibit characteristics shared by carbon dots (CDs) and graphene. Zero-dimensional carbon-based materials, CDs and GQDs, possess distinctive physicochemical characteristics linked to quantum size and edge effects. CDs are predominantly produced through bottom-up approaches and exhibit a spherical morphology of up to 10 nm. In contrast, GQDs are commonly obtained from substances in which the sp2 carbon atoms are arranged in a graphene-like configuration [194]. The large surface-to-volume ratio and a graphene structure facilitate functionalization at a considerable number of sites on the graphene surface. Covalent bonding and conjugation of antibodies, proteins, nucleic acids, and polymers to the graphene surface of GQDs have resulted in the fabrication of biosensors that are exceptionally sensitive and selective, capable of identifying biomarkers linked to a wide array of cancer types [195]. For the early and sensitive detection of cancer biomarkers, nanomaterial-based biosensors containing GQDs as a sensing platform show great promise due to their unique chemical and physical properties, large surface area, and ease of functionalization with various biomolecules capable of recognizing relevant cancer biomarkers. It has been demonstrated that optical, electrochemical, and chemiluminescent biosensors based on GQDs enable the accurate diagnosis of various cancer diseases and the assessment of anticancer therapy efficacy [195]. Using a specialized PCL–gelatin core-shell NFM, GQDs were labeled with an antibody specific for CDE isolated from blood serum. The membrane demonstrated exceptional sensitivity in isolating EXOs from a complex mixture like serum. Furthermore, the isolated EXOs were detected with great sensitivity by the GQD-antibody complex. The conclusive outcomes validate the applicability of this technique for determining the concentration of a specific analyte within a mixture [196]. Phosphorodiamidate morpholino oligomers (PMO)-functionalized RGO FET biosensors for ultrasensitive detection of exosomal microRNAs are shown by investigators. Following the fabrication of the RGO FET sensor, a layer of polylysine (PLL) film was applied onto the surface of the RGO. The synthesis of a GQDs-PMO hybrid, which was covalently bound to the PLL surface, facilitated the identification of exosomal microRNAs. With a high degree of specificity and a detection limit of 85 aM, the method was successful. In addition, exosomal miRNAs were detected in plasma samples by the FET sensor, distinguishing breast cancer samples from healthy samples. Researchers use GQDs to further increase the sensitivity of FET compared to other techniques, thereby establishing it as a potential tool for early breast cancer detection [197].

Au–carbon quantum dots (GCDs), an innovative fluorescent nanomaterial, exhibit remarkable biocompatibility and can be readily synthesized using a microwave-assisted technique. By their diminutive dimensions and distinctive optical characteristics, GCDs are viable imaging modalities for biological targets, including cells, EXOs, and various organelles. GCDs were utilized for EXO fluorescence imaging in this investigation. EXO-specific nanoprobes are generated by attaching tumor-specific antibodies to GCDs. EXOs can be labeled by the nanoprobes via immunoreactions, enabling fluorescent imaging of the EXOs. The cells can internalize EXOs labeled with nanoprobes when the two are incubated. Experiments conducted within cells confirmed that most EXOs were transported to lysosomes via endocytosis. The GCDs have no discernible impact on the process of EXO uptake or the intracellular distribution of EXOs. The experimental outcomes effectively illustrated that the nanoprobe could be utilized to investigate the intrinsic intracellular characteristics of EXOs derived from tumors. The potential of the GCDs-based nanoprobe in investigating cellular processes associated with EXOs, including cancer metastasis, is substantial [198] (Table 2).

**Table 2:** Nonmetallic NPs-based optical (nano) biosensors for detecting EXOs in cancer

Optical biosensors	Nanoparticles	Cancer	Sensitivity (LOD)	Explain	Ref
DNA nano-biosensor	GO	Lung cancer	25 fmol/µL	The approach relies on the adsorption of fDNA labeled with FAM onto GO, which subsequently inhibits the fluorescence of FAM. The formation of dsDNA from its cDNA led to fluorescence recovery from FAM through desorption and release from GO.	[187]
Transistor Biosensor	RGO	Prostate cancer (PCa)	33 particles/µL	Researchers, develop an electrical and label-free method to directly detect high-sensitivity exosomes (EXOs) based on a reduced graphene oxide (RGO) field effect transistor (FET) biosensor. In addition, the FET biosensor was employed to detect EXOs in clinical serum samples, showing significant differences in detecting healthy people and prostate cancer (PCa) patients.	[76]
A DNase I enzyme-assisted fluorescence	GO	colorectal cancer (CRC)	2.1 × 10^4^ particles/µl	Scientists devised a DNase I enzyme-assisted fluorescence amplification technique to detect CRC EXOs via interactions between graphene oxide (GO) and DNA aptamers (CD63 and EpCAM aptamers). As a result of the digestion of single-stranded DNA aptamers on the EXO surface by the DNase I enzyme, the EXOs were capable of interacting with a more significant number of fluorescent aptamer probes, which led to an augmentation in signal amplification.	[188]
Multi-Quantum Dots-Embedded Silica-Encapsulated Nanoparticle-Based	Silica-encapsulated	-	117.94 EXO /µL	Researchers, demonstrates that HFF EXOs can be detected using CD63 Ab-conjugated M–QD–SNs in the fLFA with test strips. M–QD–SNs with multiple QDots on the surface represented high PL intensity without a significant decrease in QY compared with previous studies using existing hydrophobic QDots. Furthermore, the wide absorbance range and strong fluorescence signals were suitable for use with a commercial strip reader.	[190]
A fluorescent biosensor	CdTe@MPA	Breast cancer	0.027 U/µL	The results show that EXOs from HER2-positive breast cancer patients exhibited a five times higher level of HER2 expression than healthy controls. Exosomal HER2 showed strong diagnostic power for HER2-positive patients, with the area under the curve of 0.969.	[191]
PMO-graphene quantum dots-functionalized field-effect transistor biosensor	GQDs	Breast cancer	85 aM	Researchers report PMO- GQDs-functionalized RGO FET biosensors for ultrasensitive detecting exosomal micro-RNAs. GQDs-PMO hybrid was prepared and covalently bound to PLL surface, enabling detection of exosomal miRNAs.Compared with other methods, researchers used GQDs to improve the sensitivity of FET further, making it a potential tool for early diagnosis of breast cancer.	[199]

### EXOs detection by using metal nanoparticles-modified optical biosensors in cancer

Optical biosensors demonstrate promising medical sensing capabilities when various NPs are incorporated into their architecture. The widespread application of AuNPs, AgNPs, bimetallic NPs, and magnetic NPs in the fabrication of sensing instruments is due to their distinctive optical properties and biological compatibility [200]. Furthermore, due to their facile functionalization and distinctive optoelectronic characteristics, metal NPs have emerged as exceptional analytical scaffolds. As a result, numerous analytical methods have been devised to determine the plasmonic characteristics of these NPs. Because their LSPR bands fall within the visible spectrum, Au, Ag, and copper (Cu) are appealing contenders for optical technologies. This is in stark contrast to transition metals, whose plasmon bands are situated in the UV region. However, due to Cu’s susceptibility to oxidation, most research on mNPs has concentrated on Au and Ag [201].

Furthermore, extremely small metal nanoclusters (e.g., Au, Ag, and Cu) exhibit remarkable fluorescent characteristics. To quantify EXOs, Ye’s group proposed a copper-mediated signal-amplified method [202]. The citation EXOs were captured using cholesterol-modified MBs in this study via the hydrophobic interaction between the cholesterol group and the lipid membrane. EXOs were labeled with CuO NPs modified with CD63 aptamers, and Cu^2+^ ions were liberated via acidolysis after the magnetic separation. A considerable quantity of liberated Cu^2+^ ions could be reduced by utilizing poly(thymine) as the template to form fluorescent Cu nanoclusters [156]. An economical method was suggested by researchers to directly capture and rapidly monitor EXOs using a multifunctional SERS substrate comprised of Cu_2_O–CuO nanowires synthesized via a straightforward thermo-oxidative process, followed by sputtering with Ag NPs. This reticulated substrate, composed of interlaced one-dimensional nanowires, was exceptionally well-suited for the collection and recognition of EXOs. Due to the uniform physical deposition, the electromagnetic regions exhibiting high density were notably dispersed across the nanowires. This characteristic enabled the nanowires to possess a robust SERS property, as evidenced by the signal reproducibility (13%) and enhancement factor (EF) of 3.3 × 10^8^. In addition, the effective charge transfer facilitated a further improvement in SERS performance due to the presence of Cu_2_O–CuO heterojunctions, thereby instigating a substantial chemical enhancement effect. In conclusion, clinical validation of the proposed immunosensor using serum samples from patients with prostate cancer demonstrated its high potential for rapid cancer screening and diagnosis [203].

#### Gold nanoparticles-based optical biosensors for detection of EXOs in cancer

In biomedical research, AuNPs have garnered considerable interest because of their unique optical characteristics. The AuNPs are stable, biocompatible, surface-controlled, and simple to fabricate; they also possess surface plasmonic properties. Optical biosensors based on AuNPs are capable of significantly enhancing these devices’ sensitivity, specificity, resolution, depth of penetration, contrast, and velocity. The biosensors based on AuNPs are distinguished by their luminescence, LSPR, and SERS. Protein biomarkers may be detectable at a low detection level using biomarkers based on AuNPs [204]. The aptamer exhibited binding affinity towards the target protein and unlocked the motor strand in the presence of EXOs, thereby instigating the DNA motor process. The motor strands traversed the track autonomously, facilitated by restriction endonuclease, resulting in the liberation of numerous fluorescent molecules. To extinguish the luminescence, Au nanorods (AuNRs) possess a plasmonic extinction band tunable about the aspect ratio. Chen et al., for example, described a straightforward paper-supported biosensor employing AuNRs to inhibit the luminescence of upconversion NPs (UCNPs) via luminescence resonance energy transfer. The aptamer sequence toward the CD63 protein is separated into two flexible ssDNA fragments (CP and DP), each with a unique sequence. Using Schiff base formation, branched polyethylenimine (PEI)-modified UCNPs and CP were immobilized on the surface of filter paper. CD63, present on the surface of EXOs, promoted the amalgamation of DP and CP into the intact aptamer tertiary when EXOs were introduced; this interaction ultimately led to the dissolution of AuNRs and UCNPs. Considering the possibility of LRET, the distance between AuNRs and UCNPs was reduced. Nevertheless, the CD63 prevented any interaction between two fragments, thereby precluding the occurrence of the LRET [155].

SPRi has garnered considerable interest due to its distinctive attributes, including the ability to capture the resultant refractive index change in real time while monitoring the reaction that occurred on the device. SPRi detection employs DNA probes or antibodies modified on the surface of an Au chip to form polymers with increased mass, thereby capturing the targets via specific chemical interactions. The self-assembly of DNA chains, which results in the formation of multi-layered porous hydrogels, provides an ample number of loading sites for AuNPs, thereby enhancing the signal response of SPRi detection via the LSPR effect. Of the hydrogels’ porous structure and substantial specific surface area, AuNPs are incorporated within them via Au-S bonds. This not only prevents agglomeration but also enables efficient SPR signal amplification. Furthermore, an innovative label-free, real-time SPRi biosensing approach was developed by the researchers to detect prostate cancer EXOs effectively. This strategy utilized H-Au assembled from multi-layered porous hydrogels and AuNPs. Many AuNPs were systematically and accurately positioned along the nanoscale grids of hydrogel spheres. As a result, the mass cumulative SPRi response of the hydrogels and the LSPR effect of the AuNPs were significantly enhanced. Specifically, scientists achieved the sensitive detection of EXOs in clinical samples, establishing the strategy’s promising clinical applicability. For the precise and sensitive detection of EXOs derived from prostate cancer cells, a label-free and real-time SPR imaging biosensor based on the hydrogel-AuNP supramolecular sphere (H-Au) has been developed. By incorporating the signal amplification impact of the mass cumulative hydrogel and the LSPR effect of AuNPs using a highly specific aptamer, the SPRi biosensor was capable of detecting EXOs and had a linear range of 1.00 × 10^5^ to 1.00 × 10^7^ particles/mL, with a detection limit of 1.00 × 10^5^ particles/mL. Significantly, the biosensor demonstrated remarkable feasibility for human serum analysis, as evidenced by the robust correlation between the SPRi signal and the t-PSA value assessed by the clinical chemiluminescence immunoassay. Moreover, it holds considerable promise for applications in bioanalysis and disease diagnosis [205].

Blood EV-derived integrin 6β4 has been discovered to directly promote the progression of breast cancer, thereby facilitating the detection of the disease. Antibodies obscured by an abundance of background EVs impede protein detection despite the size and heterogeneity of EVs. Thus, novel instruments with high selectivity and minimal interference for the efficient detection of EVs are highly desired. For the detection of integrin 6β4 derived from EVs, a novel Ag-coated Au nanorod SERS probe, referred to as Au@Ag@IDA-B/4MSTP, was developed in this study. This probe is based on a DNA aptamer. By validating the Au@Ag@IDA-B/4MSTP probes with EVs derived from cell culture, an LOD of 23 particles/µL was determined for EV detection. It was additionally validated that this instrument could simulate the actual condition of cancer using a mouse model of subcutaneous tumors and pulmonary metastases. The test performed a straightforward Raman analysis procedure using 10 L of blood plasma and obtained a sensitivity of 85.7% and specificity of 83.3%. Furthermore, the researchers’ methodology attains a streamlined approach that accelerates the detection process. The findings presented in this study illustrate the effective identification capabilities of Au@Ag@IDA-B/4MSTP probes for EV integrin α6β4. Investigators demonstrated that this non-invasive methodology may hold promise as a tool for the timely detection of breast cancer progression [206].

An additional investigation demonstrates that the sensitive identification of cancer-associated exosomal microRNAs has tremendous diagnostic potential. In this study, a ratiometric fluorescent biosensor was developed for the sensitive detection of CRC-associated exosomal miR-92a-3p. The biosensor utilized self-assembled fluorescent Au NPs and duplex-specific nuclease (DSN)-assisted signal amplification. The biosensing system utilizes hairpin DNA modified at both ends with the fluorescent pigment Atto-425 and sulfhydryl. The hairpin DNA is conjugated to fluorescent Au NPs via Au–S bonds, which causes the atto-425 to be quenched. The formation of a miR-92a-3p/DNA heteroduplex, which involves the opening of the DNA hairpin, induces the specific cleavage of DSN for the DNA contained within the heteroduplex. Consequently, Atto-425 recovers the fluorescence emission and departs from the fluorescent Au NPs. In forming a signal amplification cycle, the released miR-92a-3p can hybridize with another hairpin DNA, resulting in a more pronounced fluorescence recovery of Atto-425. A ratiometric fluorescent system is formed by the stable fluorescence of Au NPs and the variable fluorescence of Atto-425, which both indicate the concentration of miR-92a-3p. By identifying healthy individuals from CRC patients through the detection of miR-92a-3p extracted from clinical EXO samples, this biosensor demonstrates remarkable specificity. It holds promise for diagnosing CRC [207].

**Fig. 7:**
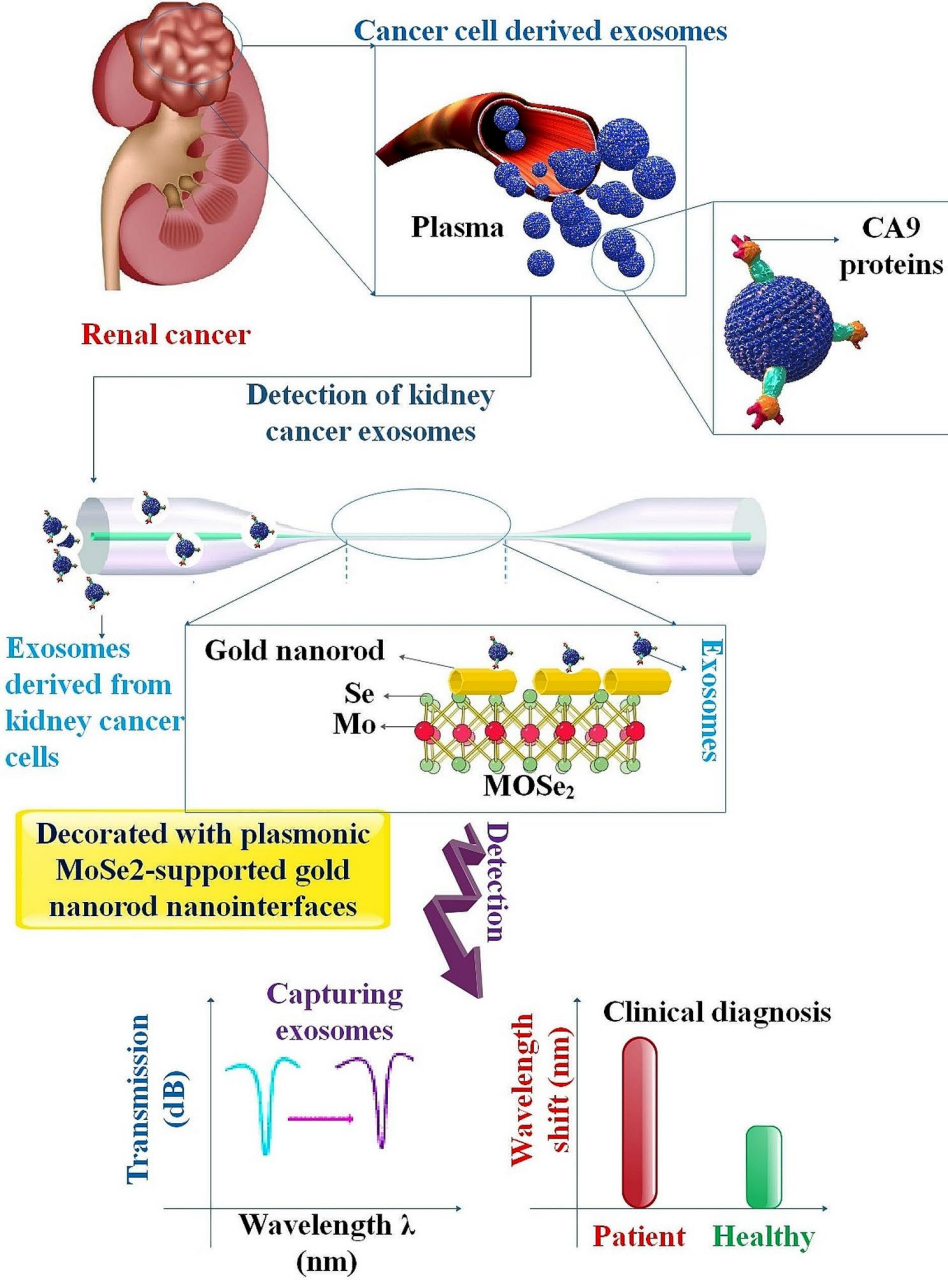
The microfiber with high specificity can successfully differentiate pathological plasma and healthy controls, exhibiting up-and-coming clinical applications in renal cancer diagnosis and prognosis. This work opens up a new approach for the in-situ detection and quantification of EXOs with ultrahigh sensitivity in early clinical screening and diagnosis [208]

Despite the widespread proposal of numerous conventional methods for detecting tumor EXOs, their limited sensitivity and specificity pose substantial obstacles to their application in cancer clinical diagnosis and prognosis. A proposed solution to the issues mentioned above is the integration of MoSe_2_-supported Au nanorods into an optical microfiber. To synchronize the strong LSPR of the nanointerfaces on the optical microfiber with the operational wavelength of the silica optical microfiber in the telecommunication band, this study proposes the use of Au nanorods featuring a high aspect ratio of around 10:1. The sensor demonstrates the ability to detect clear cell renal cancer EXOs over a broad concentration range of 100 particles/mL to 10^8^ particles/mL by utilizing the interaction between the excited LSPR effect and the evanescent field of the optical microfiber. Furthermore, it achieves an extremely low LOD of 9.32 particles/mL, significantly lower than the current state-of-the-art methods. Moreover, the microfiber, which possesses a high degree of specificity, effectively distinguishes between pathological plasma and that of healthy controls. As a result, its clinical applications in the diagnosis and prognosis of renal cancer appear extremely promising. This study introduces a novel methodology for the in situ quantification and detection of EXOs in early clinical screening and diagnosis with ultrahigh sensitivity [208] (Fig. 7).

Circulating EXOs (crEVs) have garnered growing attention in the field of non-invasive cancer diagnosis and treatment response surveillance, according to another study. As a biomimetic probe for crEVs, cancer cell membrane-functionalized AuNPs (CCM@AuNPs) containing overexpressed Tenascin-C (TNC) were synthesized in this article. The identification of crEVs incorporating fibronectin 1 (FN1) may be achieved via a distinct interaction between FN1 and TNC. To optimize the SPR signal and reduce nonspecific interactions, CCM@AuNPs were affixed to the Au chip surface, thereby ensuring the accuracy and specificity of the sensor. The detection of ultra-sensitive crEVs was accomplished using CCM@AuNPs, which exhibited a linear range of 3 × 10^4^∼3 × 10^7^ particles mL^− 1^ and a low detection limit of 18.1 particles/mL^− 1^. This biomimetic approach presents novel prospects for clinical, non-invasive cancer diagnosis through its straightforward and immediate identification of crEVs. Furthermore, a natural receptor probe-based biosensing platform was developed by the researchers to detect crEVs via the interaction between FN1 and cancer cell surface overexpressed TNC. AuNPs that were functionalized with the cancer cell membrane (CCM) were produced through the process of encapsulating the AuNPs with the cancer cell membrane. The anchoring of CCM@AuNPs to the chip surface is facilitated by para-sulfonatocalixarene (pSC4) via host-guest recognition and hydrophobic cavities. AuNPs functionalized with cancer cell membranes are resistant to interference proteins. And FN1, which is typically overexpressed in crEVs, recognizes explicitly the TNC protein on the membrane of cancer cells. The SPR angle underwent substantial alterations due to the specific binding of CCM@AuNPs to FN1 when crEVs were present. Hence, the biomimetic sensor platform represents an innovative approach to detecting crEVsb with enhanced specificity and sensitivity [209].

B bladder cancer (BC) is one of the most prevalent malignancies worldwide, with high morbidity and mortality, according to another study. A fluorescent biosensor was introduced in this study, which utilized a combination of inorganic nanoflares and a DNAzyme walker to detect BC exosomal microRNAs concurrently. The biosensor was developed using AuNP that had been modified with DNAzyme strands and carbon dot (CD)-labeled substrates (AuNP@CDs inorganic nanoflares-DNAzyme, APCD). DNAzyme was activated in the presence of target miRNAs; it cleaved the CD-labeled substrates and moved autonomously along the AuNP, thereby facilitating fluorescence recovery. The APCD biosensors exhibited exceptional sensitivity and specificity due to their design and functionality. The LOD for a solitary miRNA was determined to be in the femtomolar range, and the linear range extended from 50 fM to 10 nM. In addition, concurrent qRT-PCR analysis of exosomal miR-133b and miR-135b associated with BC was performed on clinical serum samples; this finding supports the potential of this method in diagnosing BC and other malignancies [210].

#### Silver nanoparticles-based optical biosensors for detection of EXOs in cancer

The feasibility of identifying specific EXO-like vesicles (ELVs) was illustrated by scientists through the application of SERS to analyze their molecular signatures after the functionalization of ELVs with AuNP stabilized with (dimethylamino)pyridine (DMAP). While this approach successfully differentiated ELVs from various cellular sources, the SERS spectra yielded suboptimal results owing to the substantial background introduced by the DMAP stabilizing molecules on the AuNP surface. A core-shell NP (Au@AgNPs) can be formed directly at the ELV surface by overgrowing in situ the ELV-attached AuNPs with an Ag layer, as demonstrated in this study. This method eliminates interfering SERS signals from stabilizing molecules at the AuNP surface. Therefore, this methodology signifies the initially established approach to produce unobstructed SERS spectral signatures of intricate biological structures, devoid of the influence of linker molecules—which are indispensable for facilitating the association of the plasmonic NP with the ELV surface and guaranteeing its colloidal stability. By employing core-shell plasmonic NPs as the SERS substrate in this novel approach, near-field enhancements were significantly more significant than in previous methods, leading to SERS spectra with an enhanced signal-to-noise ratio. This enabled scientists to distinguish between vesicles originating from B16F10 melanoma cells and red blood cells (RBCs) with a sensitivity and specificity greater than 90%, which was previously unattainable. Significantly, by utilizing the enhanced near field, the acquisition time was diminished by a factor of 20 compared to previously documented approaches. This advancement paves the way for identifying single ELVs with high throughput without the need for labels. Due to the presence of molecules originating directly from the progenitor cell, these vesicles have garnered attention as potential biomarkers for the timely identification and surveillance of a wide range of ailments, including cancer [211]. Bin-Cheng Yin et al. reported an additional investigation wherein they utilized MBs and a SERS aptasensor composed of Au-Ag-Ag core-shell-shell nanotrepangs (GSSNTs) to identify three distinct types of cancer-related EXOs. The sensing platform is comprised of two components: a capture probe and a SERS detection probe, both of which are embellished with various biomolecules. To construct SERS detection probes, functionalized GSSNT nanotags were affixed with specific Tris(2-carboxyethy1) phosphine (TCEP)-activated linker DNAs (L-PSMA for aptamer of LNCaP EXOs, L-Her2 for aptamer of SKBR3 EXOs, and L-AFP for aptamer of HepG2 EXOs). In contrast, three Raman dyes—2-Mpy, 4-ATP, and NTP—were utilized to decorate the synthesized GSSNT nanotags, resulting in SERs-active nanotags. Conversely, the multiplex capture probes were constructed through the co-assembly of streptavidin-modified MBs and aptamer DNAs (Apt-PSMA was utilized to assemble LNCaP EXOs, Apt-Her2 was employed to assemble SKBR3 EXOs, and Apt-AFP was used to assemble HepG2 EXOs). After these two components were incubated together, the SERS sensor that was proposed was established. The SERS sensor detected and captured the three target EXOs through affinity adsorption after a mixture of three EXOs was introduced into the sensor. The aptamer sequences on the capture probes then liberated the SERS detection probes into the mixture. Thus, it was demonstrated that the aforementioned SERS-based sensor can multiplexing and detecting three cancer-related EXOs simultaneously [212, 213].

#### Titanium nanoparticles-based optical biosensors for detection of EXOs in cancer

Compared to Au and Ag, titanium nitride (TiN) is an exceptional alternative plasmonic supporting material due to its adjustable plasmonic characteristics in the visible and NIR regions. Nevertheless, reports of label-free SPR biosensing utilizing TiN are infrequent owing to inadequate protocols for surface functionalization. This study presents biotinylated antibody-functionalized TiN (BAF-TiN) for label-free biosensing applications requiring high performance. EXOs ranging in size from 30 to 200 nanometers (nm) were quantified using the BAF-TiN biosensor on a human glioma cell line. It has been determined that the BAF-TiN biosensor has a LOD of 4.29 × 10^− 3^ µg.mL^− 1^ for CD63, an EXO marker, and 2.75 × 10^− 3^ µg.mL^− 1^ for EGFR variant-III, a mutant protein specific to glioma. In summary, the BAF-TiN biosensor exhibits considerable promise in identifying cancer biomarkers, such as exosomal surface proteins, by capitalizing on the biocompatibility, stable nature, and exceptional label-free sensing capabilities of TiN. With their prospective biosensing structure and alternative plasmonic medium, BAF-TiN biosensors are anticipated to find applications in the pharmaceutical industry and various biomedical disciplines, including disease diagnosis, immunotherapy, and pathogen detection. As a result of their excellent biocompatibility and chemical stability, BAF-TiN biosensors may also be used to monitor in vivo biotargets in real time [214].

Researchers developed a PDIG film capable of perceiving multiple signals from a single stimulus in an investigation. The thermochromic and thermosensitive characteristics of PDIG are attributed to the presence of deep eutectic solvents (DES) and cobalt(II) chloride. In a more specific manner, the PDIG functioned as the interface for recognition in conjunction with a bipolar electrode (BPE) that demonstrates exceptionally responsive conductivity and color-to-temperature variations. This response is triggered by the light-harvesting probe TiO_2_@CNOs, which was introduced via proximity hybridization assay. The PDIG generated colorimetric, photoacoustic, and electrochemiluminescent signals, which were utilized to detect EXOs associated with CRC. It is anticipated that this research will pave the way for further investigation into the multisignal amplification strategy of BPE, expand the utilization of BPE in biological analysis, and offer fresh perspectives on the development of exceptionally information-sensitive components that guarantee multimodal coupling for the detection of cancer-specific EXOs [215]. An additional investigation devised a SERS immunoassay to detect ExoPD-L1 with exceptional sensitivity. This assay capitalized on the non-selective affinity for MXene and the specific recognition capability exhibited by Au@MPBA@SiO_2_-pep SERS tags. Extensive ligation of Ti-O and Ti-F to the phospholipid membrane of EXOs on the Au chip-deposited MXene permits the indiscriminate capture of EXOs. A SERS immunoassay was designed to detect ExoPD-L1 with exceptional sensitivity. This detection method capitalized on the specific recognition capability of Au@MPBA@SiO_2_-pep SERS tags and the nonselective recognition ability of MXene. Extensive ligation of Ti-O and Ti-F to the phospholipid membrane of EXOs on the Au chip-deposited MXene permits the indiscriminate capture of EXOs [216].

#### Magnetic nanoparticles-based optical biosensors for detection of EXOs in cancer

**Fig. 8:**
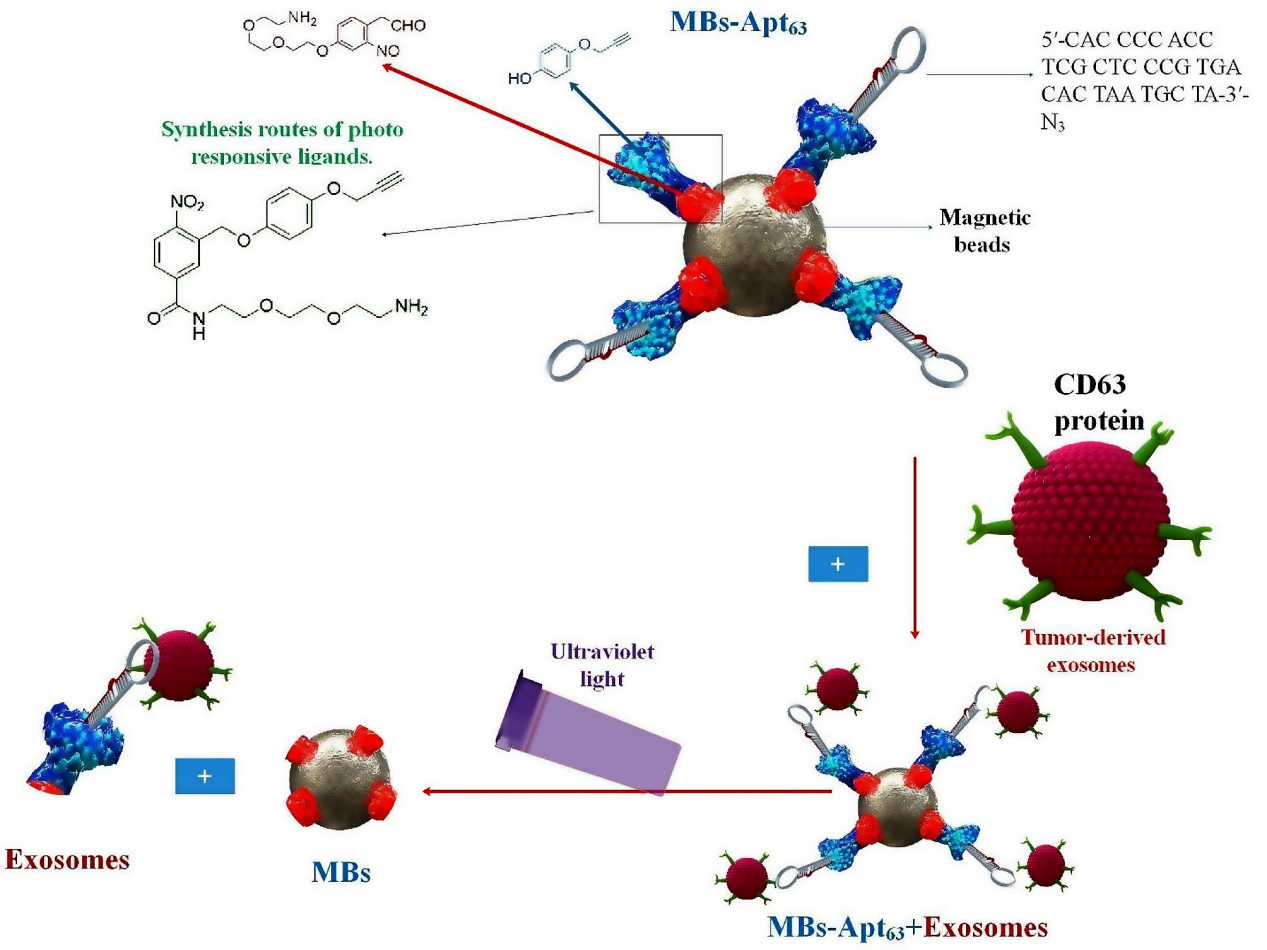
Exosome binding and dissociation using UV-responsive nanomagnetic beads, shown schematically. CD63 is a component of tetraspanins, regarded as dependable surface markers for exosomes (EXOs). It has proved to be a biomarker for breast cancer EXOs and performs crucial functions in membrane transport. Researchers’ magnetic bead system can selectively bond to the CD63 protein on EXOs following incubation with serum from rodents bearing breast cancer. Subsequently, the target EXOs can be isolated using an external magnetic field. To elute EXOs that are both structurally and functionally intact from magnetic particles, light excitation would be regulated. By employing this nanodevice, a regulated approach is taken to isolate EXOs from serum, thereby optimizing the EXOs’ utility in liquid biopsies and clinical investigations. Simultaneously, the optical control strategy-based magnetic particle sorting method provides a new platform for future EXO detection of liquid homogeneous substances [217]

EXOs derived from tumors are regarded as crucial biomarkers within the domain of liquid biopsy. On the contrary, traditional methods of separation, including ultracentrifugation, co-precipitation, and column chromatography, are incapable of isolating samples at a high throughput. Furthermore, the steric effect of MBs in conventional immunomagnetic separation techniques diminishes the optical detection sensitivity of EXOs. Researchers present a novel and straightforward nanoplatform in this study that enables spatiotemporally controlled EXO extraction and elution via light-activated cargo release and magnetic separation. Covalent aptamers and photoresponsive groups (nitrobenzyl group) modify MBs in this system in a manner that is compatible with CD63, an exosomal surface-specific protein that is highly expressed. By performing proteomic analysis and morphological characterization of EXOs obtained from a xenograft tumor model in nude mice and a cell model, researchers’ novel magnetic bead system demonstrated superior performance in serum EXO extraction compared to existing ultracentrifugation methods. This superiority was observed regarding extraction time, yield, and the proportion of populations exhibiting high CD63 expression. 2-nitrobenzene is utilized as the surface ligand of the MBs in this approach; the beads’ susceptibility to ultraviolet light enables spatiotemporal regulation of EXO separation with minimal disruption to the serum composition. Practically speaking, it necessitates a reduced time investment, a smaller sample volume, and the absence of sophisticated instruments. Simultaneously, EXOs in the actual liquid environment can significantly enhance the efficiency of the labeled enzyme and substrate reaction unaffected by the interference of MBs. Furthermore, the effect of MBs obscuring one another will be eradicated. Hence, by employing this approach, the sensitivity of successive detections ought to be enhanced [217] (Fig. 8).

An additional research endeavor is to utilize tumor-derived circulating EXOs (TDEs) as noninvasive and informative biomarkers. Nevertheless, the quantitative detection of TDEs remains a formidable task. To develop a swift and sensitive platform for analyzing TDEs, the authors of this study developed a microfluidic magnetic detection system (µFMS) mediated by a DNA tetrahedral-structured probe (TSP). To fabricate magnetic nano-report probes (MNRs), CD63 aptamer-modified Fe_3_O_4_ magnetic NPs (MNPs) were developed. To manufacture the microfluidic circuits, glass was functionalized with aldehyde groups modified with DNA TSP, and a PDMS substrate featuring serpentine microchannels was incorporated. Utilizing an induction coil-based magnetic detector, the magnetic signal was quantified. The FMS system exhibited a linear dynamic range of 1.98 × 10^3^–1.98 × 10^7^ particles/mL when used for TDE assays. Additionally, it had a LOD of 1.98 × 10^3^ particles/mL in PBS. The lack of a statistically significant distinction in TDE detection between the synthetic serum and PBS suggests that the developed µFMS system can effectively analyze TDEs in complex biological systems. Considering cost, reaction time, and operation procedure, this FMS exhibits promise as a clinical point-of-care diagnostic instrument for therapeutics and cancer diagnosis. Furthermore, the researchers demonstrated that the detection outcomes of TDEs did not differ significantly between the simulated serum system and the PBS buffer. In contrast to alternative approaches for identifying TDEs, such as electrochemical or optical methods, µFMS demonstrated significant improvements in detection time and sensitivity. As a result, the µFMS system that investigators have developed can serve as a practical POCT instrument and may offer an exceptional alternative for noninvasive liquid biopsy [218].

A separate investigation created a dual-aptamer-based photoelectrochemical (PEC) biosensor that does not require immobilization. This sensor is designed to enrich and quantify cancer EXOs. It operates via nucleic acid-based recognition and signal amplification, utilizing photoactive bismuth oxyiodide/Au/cadmium sulfide (BiOI/Au/CdS) composites. The deoxyribonucleotidyl transferase (TdT) enzyme-assisted polymerization process would be initiated in this biosensor upon two aptamers recognizing the EXO. This would result in the accumulation of ALP on the Fe_3_O_4_ surface. Following magnetic separation, ALP could catalyze the production of ascorbic acid (AA) in the form of an electron donor, thereby instigating the subsequent redox cycle reaction that enhanced the signal. Additionally, since the steric hindrance effect was avoided throughout the aforementioned steps, recognition and signal amplification efficiency would be higher than with the heterogeneous technique. Consequently, the suggested PEC biosensor demonstrated remarkable sensitivity and selectivity in enriching and identifying cancer EXOs. The biosensor’s linear range was calculated to be 21 particles·µL^− 1^, and its detection limit was 1.0 × 10^2^ particles/µL^− 1^ to 1.0 × 10^6^ particles/µL^− 1^. Thus, the suggested PEC biosensor has a lot of potential for use in other bioassay applications and nondestructive early clinical cancer diagnostics by detecting tumor EXOs. By using the aptamer’s particular recognition capability and the signal amplification processes’ high efficiency, the suggested PEC biosensor demonstrated remarkable sensitivity and selectivity in the detection of CDE [219].

Around 20–25% of women with breast cancer (BC) overexpress the aggressive marker human HER2, making BC a severe worldwide health concern. However, access to early diagnosis and treatment differs throughout nations. For usage in low- and middle-income countries (LMICs), a low-cost, equipment-free, and user-friendly polydiacetylene (PDA)-based colorimetric sensor is developed to detect HER2-overexpressing cancer. The initial step in creating PDA NPs is thin-film hydration. Then, they are successively coupled to HER2 antibodies and hydrophilic magnetic NPs. While retaining PDA’s optical properties, the synthetic HER2-MPDA may be focused and separated by a magnetic field. By changing the molecular structure of the PDA backbone, the selective binding of HER2 antibody in HER2-MPDA to HER2 receptor in HER2-overexpressing EXOs results in a blue-to-red color shift. HER2-overexpressing EXOs may be simultaneously separated and detected by this colorimetric sensor. In the culture medium of HER2-overexpressing BC cells and urine samples from HER2-overexpressing BC mice models, HER2-MPDA can identify HER2-overexpressing EXOs. Through magnetic separation, it can only identify and isolate HER2-overexpressing EXOs, and in LMICs, its detection limit is determined to be BC. Particle size: 8.5 × 10^8^ mL^− 1^. This colorimetric sensor may diagnose HER2-overexpressing patients at the point of care [220].

### EXOs detection by using Silicon nanoparticles-modified optical biosensors in cancer

Expensive equipment, low sensitivity, and complex procedures continue to be obstacles to conventional EXO detection methods, according to this study. By their rapid response, high sensitivity, and label-free detection, FETs not only constitute the most indispensable electronic component in the contemporary microelectronics industry but also exhibit tremendous potential for biomolecule detection. To detect EXOs electrically and without the need for labels, the authors of this study proposed a Si nanowire field-effect transistor (Si-NW Bio-FET) device that had been chemically modified with specific antibodies. The Si-NW FETs were produced using conventional microelectronic techniques and nanowires measuring 45 nm in width. They were then encapsulated in a microfluidic channel made of polydimethylsiloxane (PDMS). The nanowires underwent additional modifications utilizing the CD63 antibody to fabricate a Si-NW Bio-FET. A successful demonstration was conducted on the electrical and label-free detection of EXOs utilizing the Si-NW Bio-FET that was developed. The LOD for this detection was determined to be 2159 particles/mL. In contrast to alternative technologies, Si-NW Bio-FET presents a distinctive approach in this research endeavor to quantify and detect EXOs in real time without labeling. This suggests that the technology may have utility in the realm of cancer early detection. In conclusion, for the electrical and label-free detection of EXOs, scientists created a Si-NW Bio-FET functionalized with a CD63 antibody that exhibited exceptional sensitivity. As a result of the positively charged target analyte, the Si-NW Bio-FET current was diminished. As EXO concentration increased, the threshold voltage of the transfer characteristic curves shifted to the left. For A549 EXOs, the Si-NW Bio-FET’s LOD was 2159 particles/mL, which was greater than the LOD for several conventional electrochemical techniques. Furthermore, the Si-NW Bio-FET can track the real-time variation in EXOs. As the concentration of EXOs increased, the real-time current dropped. Future applications of this research, which offers a novel CMOS-compatible method for sensitive, label-free, real-time EXO detection, include clinical diagnosis and real-time monitoring [221].

The objective of this research endeavor was to develop a microfluidic electrochemical immunosensor capable of accurately and sensitively measuring epithelial growth factor receptor (EGFR) levels in extracellular vesicles (EVs) extracted from the plasma of patients diagnosed with breast cancer. The reaction platform of the sensor is composed of chitosan-coated SiNPs (SiNPs-CH), which are utilized in conjunction with SiNPs-CH to retain the monoclonal anti-EGFR antibody covalently within the central channel (CC) of the microfluidic device. Utilizing UV–visible spectroscopy, energy dispersive spectrometry (EDS), NTA, and TEM, the synthesized SiNPs-CH were characterized. EGFR was quantified using horseradish peroxidase (HRP)-conjugated anti-EGFR in a direct sandwich immunoassay. On an Au electrode sputtering, the enzymatic product, benzoquinone, was identified by reduction at -100 mV. The amount of EGFR in human serum samples was proportionate to the observed current. There was a linear range of 0 ng mL^− 1^ to 50 ng mL^− 1^. The within- and between-assay coefficients of variation were below 6.25%, and the detection limit was 1.37 pg mL^− 1^. Ultimately, the new technique was used to examine plasma samples from 20 healthy donors and 30 patients with early-stage breast cancer. EGFR levels in EVs (EVs-EGFR) were shown to have greater sensitivity and specificity than normal blood indicators like CEA and CA15.3, and they were also considerably higher than in the healthy control group. The EGFR tumor status, tumor size, and pathological grade correlate with the EVs-EGFR concentration. In summary, plasma EVs can be utilized in the proteomic characterization of cancer, provided that the technique employed has adequate sensitivity. This is particularly true for immune-electrochemical nanosensors with increased reaction surface area. A microfluidic immunosensor has been developed to enable the quantification of EGFR in samples of electronic vesicles. The analytical technique utilized by the researchers is founded upon the covalent immobilization of monoclonal anti-EGFR antibodies onto SiNPs-CH, which are affixed to the CC of a microfluidic device. The total assay duration utilized (less than 30 min) was shorter than the 240 min reported for a commercial ELISA test reagent that is commonly used in clinical diagnosis [222] (Table 3).

**Table 3:** EXOs detection by using metal nanoparticles-modified optical biosensors in cancer

Optical biosensors	Nanoparticles	Cancer	Sensitivity (LOD)	Explain	Ref
Upconversion luminescence	AuNPs	Several type cancers	1.1 × 10^3^ particles/µL	Researchers used a simple paper-supported aptasensor based on LRET from UCNPs to Au NRs for the accessible determination of exosomes (EXOs). Such an approach can reach a low detection limit of EXOs (1.1 × 10^3^ particles/µL) and effectively reduce the background signal by using UCNPs as a luminescent material.	[155]
Surface plasmon resonance biosensor	AuNPs	Prostate cancer	1.00 × 10^5^ particles/mL	Based on the hydrogel-AuNP supramolecular sphere (H-Au), a label-free and real-time surface plasmon resonance imaging biosensor has been developed for compassionate and specific determination of prostate cancer cell-derived EXOs. Most importantly, with a strong correlation between the SPRi signal and the t-PSA value measured by the clinical chemiluminescence immunoassay, this biosensor displayed excellent practicability for human serum analysis.	[205]
Ratiometric Fluorescent Biosensor	Au NPs	Colorectal cancer (CRC)	-	The stable fluorescence of Au NPs and the changing fluorescence of Atto-425 constitute a ratiometric fluorescent system reflecting the concentration of miR-92a-3p. This biosensor exhibits excellent specificity and can distinguish CRC patients from healthy individuals by detecting miR-92a-3p extracted from clinical EXO samples, showing the potential CRC diagnosis.	[207]
Optical Microfiber Decorated	MoSe2-supported gold nanorods	Renal cancer	9.32 particles/mL	Due to the interaction between the excited LSPR effect and the evanescent field of the optical microfiber, the sensor can detect clear cell renal cancer EXOs within a wide concentration range from 10^0^ particles/mL to 10^8^ particles/Ml.	[208]
Biomimetic probe	CCM@AuNPs	Non-invasive cancer	18.1 particles/mL^− 1^	Cancer cell membrane functionalized AuNPs (CCM@AuNPs) containing overexpressed Tenascin-C (TNC) were synthesized as a biomimetic probe for crEVs. crEVs containing fibronectin 1 (FN1) could be recognized through the interaction between FN1 and TNC.	[209]
Plasmonic Biosensor	Titanium nitride	Cancer biomarkers	4.29 × 10^− 3^µg mL^− 1^	Biotinylated antibody-functionalized TiN (BAF-TiN) for high-performance label-free biosensing applications. In conclusion, combining the biocompatibility, high stability, and excellent label-free sensing performance of TiN, the BAF-TiN biosensor could have great potential for detecting cancer biomarkers, including exosomal surface proteins.	[214]
Colorimetric sensing	TiO_2_@CNOs	Colorectal cancer	-	The PDIG served as the recognition interface in series with a bipolar electrode (BPE) that exhibits a susceptible color and conductivity response to temperature stimuli triggered by the light-harvesting probe TiO_2_@CNOs introduced via proximity hybridization assay triggering a rolling circle amplification strategy, resulting in the output of colorimetric, photoacoustic, and electrochemiluminescent signals for the detection of colorectal cancer EXOs.	[215]
SERS	Ti3C2TX	Breast cancer	20.74 particles/µL	This work provides new insights into the design of biosensors for EXO detection. It can serve as a replicable template for sandwich immunoassay detection for other types of sensors, including but not limited to SERS.	[216]
PEC biosensor	BiOI/Au/CdS	Cancer-derived EXOs	21 particles·µL^− 1^	This sensor is designed to enrich and quantify cancer EXOs. It operates via nucleic acid-based recognition and signal amplification, utilizing photoactive bismuth oxyiodide/Au/cadmium sulfide (BiOI/Au/CdS) composites. Consequently, the suggested PEC biosensor demonstrated remarkable sensitivity and selectivity in enriching and identifying cancer EXOs.	[219]
Microfluidic electrochemical immunosensor	SiNPs-CH	Breast cancer	1.37 pg µL^− 1^	A microfluidic electrochemical immunosensor capable of accurately and sensitively measuring epithelial growth factor receptor (EGFR) levels in extracellular vesicles (EVs) extracted from the plasma of patients diagnosed with breast cancer. EGFR levels in EVs (EVs-EGFR) were shown to have greater sensitivity and specificity than normal blood indicators like CEA and CA15.3, and they were also considerably higher than in the healthy control group (*p* = 0.002).	[222]
Fluorescent biosensor	AuNPs	bladder cancer (BC)	50 fM to 10 nM	A fluorescent biosensor based on inorganic nanoflares combined with a DNAzyme walker for the simultaneous detection of BC exosomal microRNAs (miRNAs). In the presence of target miRNAs, DNAzyme was activated and then cleaved the CD-labeled substrates and automatically walked along the AuNP, allowing fluorescence recovery.	[210]
SERS	Au@AgNPs	B16F10 melanoma cells and red blood cells (RBC)	sensitivity and specificity > 90%	Demonstrate that it is possible to eliminate interfering SERS signals from stabilizing molecules at the AuNP surface by overgrowing in situ the ELV-attached AuNPs with a silver layer to form a core–shell NPs (Au@AgNPs) directly at the ELV surface.	[211]

## Clinical trials

EXOs exhibit considerable potential for cancer detection due to their enrichment with biomolecules specific to cancer and their ability to identify cancer-related markers in a non-invasive, sensitive, and targeted manner. With the ongoing progression of research, EXO-based assays can fundamentally transform the field of cancer diagnosis by enabling earlier detection and enhancing patient survival and quality of life. However, it is crucial to prioritize the resolution of ethical concerns, standardization, specificity, sensitivity, and clinical validation issues to fully harness the translational capabilities of EXOs in the context of cancer detection in clinical settings [223]. Additionally, investigations have been conducted to examine EXOs extracted from human samples across various conditions. According to an analysis, a total of 215 trials have been documented, with 76 devoted to biomarker applications. 33 investigations (28.44%) to EXO therapy have been formally registered. Registration is complete for seventeen (14.66%) studies on EXOs fundamental analysis compared to six (5.17%) studies on drug delivery system trials [224].

Through searching www.clinicaltrials.gov, 76 results of the terms “cancer diagnosis” and " EXO” have been identified so far. While there hasn’t been a clinical trial using optical nanobiosensors to identify exosomal cancer biomarkers, it is anticipated that in the future, this potent technique will serve as a suitable substitute for the more traditional EXO detection techniques.

Researchers investigate the possibility of using the differential expression of exosomal protein markers to identify NSCLC patients from control participants. Plasma was extracted from 110 matched control people who were initially suspected of having cancer but were later shown to be cancer-free, and 109 NSCLC patients with advanced stage (IIIa–IV) illness. EXOs were phenotyped straight from the plasma samples using the Extracellular Vesicle Array (EV Array). EXOs were seen using a combination of biotin-conjugated CD9, CD63, and CD81 antibodies. The array, which targeted proteins linked to lung cancer, had 37 antibodies. Merely 10 µL of unpurified plasma was sufficient to identify and characterize EXOs in every sample using the EV Array analysis. The Random Forests approach was used in multivariate analysis to create a combined 30-marker model with an area under the curve of 0.83, CI: 0.77–0.90, that separated the two patient groups. With a sensitivity of 0.75 and a specificity of 0.76, the 30-marker model accurately diagnoses patients 75.3% of the time [225].

A single clinical study is being conducted to evaluate the effectiveness of EXOs in tracking patient response to lung cancer treatment. The potential use of measuring Programmed Death Ligand 1 (PDL1) mRNA expression in plasma EXOs as a means of monitoring radiation efficacy is being investigated by the clinical study NCT02869685. PDL1 is an essential receptor for naïve T-cell activation, which is critical for effectively eliminating tumor cells. Although June 2019 was the anticipated completion date of this investigation, it is still unclear how the clinical trial is progressing [20].

The significance of Glypican 1 (GPC1)-positive EXOs as liquid biopsy components is still up for dispute. Prospective research was conducted to measure the amount of GPC1-positive EXOs in the sera of patients with PDAC receiving upfront surgery, in comparison to controls, which included patients with no history of cancer and patients with pancreatic preneoplasic lesions. EVs were shown to be abundant in sera, and anti-CD63 linked magnetic beads were used to extract EXOs. Flow cytometry revealed that the GPC1-positive bead percentages in PDAC were notably more significant than those in the control group. When findings from peripheral and portal blood were pooled, diagnosis accuracy increased to 78% (sensitivity: 64%, specificity: 90%). The negative predictive value for echo-guided ultrasound fine-needle aspiration (EUS-FNA) was 80% in conjunction with it, but only 33% for EUS-FNA alone. Given that patients with GPC1-positive EXOs in their peripheral blood had worse tumor-free survival, this method is therapeutically helpful as an adjunct test to the current diagnostic techniques. NCT03032913 examined the effectiveness of GPC1 + EXOs in conjunction with CA19-9 as a PDAC diagnosis. Upon completion of this study, they discovered that GPC1 + EXOs, when combined with CA19-9, could more accurately predict PDAC than either marker alone [20, 226].

Creating the best techniques for isolating pure EXO populations is the main challenge in EXO diagnostics. Even though it takes the longest, ultracentrifugation is still the best technique for isolating and purifying EXOs. If EXOs are considered a viable option for therapeutic applications, faster and more sophisticated techniques for separating them are required. The comparative studies will only be feasible with separation methods that separate these two primary EVs into pure exosomal vs. microvesicle populations. Microvesicles themselves may be a valuable diagnostic tool to identify malignancies. There are benefits and drawbacks to these EXO detection and quantification techniques for clinical use. For instance, ELISA preparation takes some time, but once the test is complete, a positive result may be seen quickly in the colorimetric response. Nevertheless, additional biomolecules present in the patient fluid under study have the potential to contaminate ELISA. Though it requires costly equipment and has variable results, flow cytometry is capable of detecting different types of EXOs and classifying them according to species. Understanding the processes that control the heterogeneity of cancer EXOs presents another difficulty since they impact the components of exosomal cargo obtained from cancer and, therefore, the repeatability of diagnostic results. Notwithstanding these reservations, future research combining immune-affinity capture methods, proteomic analysis of exosomal surface proteins, and next-generation sequencing of exosomal RNAs and DNAs will further the use of EXOs in cancer detection [20, 23]. NTA can also not distinguish the sort of EXO it detects and necessitates costly equipment. A procedure has to be economical and employ the least amount of expensive equipment possible to be clinically helpful. Perhaps more crucially, a technique has to be consistent and conclusive—qualities that the approaches under discussion struggle to meet. So, the best approach would be one that guarantees purity quickly and does not need expensive equipment. Developing novel sensing and detecting techniques, such as electrochemical detection, microfluidics, and nano-sensors, is actively addressing these constraints [20].

Using magnetic substrates and SERS probes, the researchers in this work presented a SERS-based technique for the simultaneous multiple detection and screening of EXOs. In particular, aptamer-modified Au shell magnetic nanobeads are used to create the capturing substrates. These beads can recognize the generic surface protein CD63, which allows them to capture a wide variety of EXO types. In addition, the Raman reporter and particular aptamer for EXO targeting on Au NPs make up the SERS probes. Furthermore, three distinct types of SERS probes were created employing several SERS reporters to detect different types of EXOs simultaneously. These three types of SERS probes were combined with the capture substrates to identify certain EXO types. The relevant sort of SERS probes, the substrate, and the target EXOs may form an apta-immunocomplex when the target EXO is present, while the other non-specific SERS probes stay in the suspension. To separate the signals, they created three different kinds of SERS probes that would attach to SKBR3, T84, and LNCaP EXOs simultaneously. These probes were then utilized to capture substrates. To create a standard curve for TEX detection, the supernatant’s decrease in Raman intensities was examined. For the SKBR3, T84, and LNCa EXOs, the LOD values obtained using this approach are 32, 73, and 203 particles per microliter, respectively. Furthermore, the test was developed to identify the authentic blood samples obtained from cancer patients [227]. In SERS detection, AuNPs are the most often used materials for signal enhancement. For instance, Kwizera et al. suggested employing Au nanorods with SERS biosensors to identify proteins unique to EXO surfaces. In two hours, each device can process more than eighty pure samples with a modest concentration of EXOs (2 × 10^6^ EXOs/mL). It is notable that this approach lowers costs, increases efficiency, and shortens operating times. Ma et al. also reported on the fabrication of Au@R6G@AgAu NP, which are stable SERS probes made by attaching R6G to AuNPs and encasing them in AgAu alloy shell NPs called ARANPs. It was effectively shown that this sensor technology significantly increased sensitivity. Moreover, it may identify recurrences in individuals with NSCLC from as low as 5 µL of sample volume [21, 168, 228].

Serum microvesicles from glioblastoma patients include messenger RNA mutants/variants and microRNAs typical of gliomas. Seven of the twenty-five glioblastoma patients had serum microvesicles containing the tumor-specific EGFRvIII. Thus, using a blood test, and tumor-derived microvesicles may help cancer patients make therapy choices and give diagnostic information. EXOs generated from tumors are sources of biomarkers indicating the condition of parental cancer cells; in clinical terms, EXOs collected from blood samples of glioblastoma patients contain particular EGFR VIII [229].

Surface plasmon resonance (SPR) is a label-free, real-time, susceptible optical detection technology. High sensitivity and resolution are available from commercial prism-based wavelength/angular-modulated SPR sensors, but their cost and big footprint restrict their use in clinical settings. A novel test called nanoplasmonic EXO (nPLEX) has been created to identify exosomal proteins and diagnose ovarian cancer. However, in contrast to traditional SPR biosensors, the challenging and costly production of nanostructures restricts the wide range of applications for nanoplasmonic biosensors. Researchers have created a tiny, intensity-modulated SPR biosensor (measuring 25 cm by 10 cm by 25 cm) that utilizes a standard SPR sensing mechanism and eliminates the need for creating nanostructures. The small SPR biosensor demonstrated sensitivity of 9.258 × 10^3^%/RIU and resolution of 8.311 × 10^–6^ RIU after calibration using glycerol. Investigators have shown that the exosomal EGFR and PD-L1, two biomarkers, may be used to diagnose lung cancer using the compact SPR biosensor. A549 nonsmall cell lung cancer (NSCLC) cells have more exosomal EGFR than BEAS-2B normal cells. Using human blood samples, the compact SPR biosensor found that NSCLC patients expressed more exosomal PD-L1 than normal controls and that NSCLC patients had equal amounts of exosomal EGFR. Comparing the small SPR biosensor to ELISA, it demonstrated comparable sensing accuracy and greater detection sensitivity. It is an easy-to-use sensing platform that might be used as an in vitro cancer diagnostic test [230].

For the ultrasensitive test of prostate cancer (PC) EXOs, an optical microfibre modified with WS2-supported Au NBPs nanointerfaces is suggested. With the WS2 nanointerface in conjunction with the solid LSPR hotspots of the Au NBPs, the sensor demonstrated ultralow LODs of 23.5 particles mL^− 1^ in PBS solution and 570.6 particles mL^− 1^ in 10% serum, indicating ultrasensitive EXO detection. Two orders of magnitude less than the LOD of 23.5 particles mL^− 1^ were reported for other state-of-the-art methods. Using this sensing approach, the sensor can detect PC serum from healthy serum and correctly diagnose complete blood samples from patients by size and collecting a single bioparticle. This study offers a brand-new, compassionate approach for testing entire blood [231].

Researchers describe a microstructured optical fiber sensor for quick, sensitive, and precise EXO measurement in patient blood samples for breast cancer in another study. Using numerical simulations, it is shown that hollow-core microstructured antiresonant fibers (HARFs) may maximally amplify the signal by tightly confining light inside the fiber core, hence guaranteeing a high light-matter interaction. By taking advantage of this, an AuNPs–dsDNA assembly comprising fluorescent reporter DNA sequence, a recognizing DNA aptamer, and AuNPs is created, and it is then immobilized on the fiber wall to produce an AuNPs–dsDNA–HARF sensor. EXOs originating from cancer may be identified, caught, and quantified using dose-dependent fluorescent signals produced in the fiber channel. At the nanoliter sample level, the microfibre sensor’s improved sensitivity and specificity allow for identifying EXO particles as small as one digit. Furthermore, the suggested fiber sensor may help with accurate medication treatment monitoring by measuring changes in EXO composition. This study offers a strong foundation for EXO-based biopsy for cancer diagnosis and the forecasting of treatment results [232].

An additional study presents a divisional optical biochip that combines precise surface patterning and nanochain self-assembly to enable multiplex EXO analysis. Following their capture of target EXOs for direct visual detection, the nanochains exhibit discernible color changes induced by resonance-induced near-field enhancement. Following this, a sequence of biochips based on divisional nanochains are manufactured, each of which is conjugated with a distinct antibody via hydrophobic and hydrophilic patterns that have been designed. Due to the notable disparity in wettability, a single droplet of the sample self-divides precisely into several microdroplets, allowing for the concurrent detection of numerous target EXOs within a 30-minute timeframe. This is achieved with a sensitivity of 6 × 10^7^ particles mL^− 1^, which is approximately two orders of magnitude lower than that of the enzyme-linked immunosorbent assay. In addition to detecting trace amounts, the method exhibits exceptional semiquantitative capability in differentiating clinical EXOs from those of glioblastoma patients and healthy individuals. The simplicity, adaptability, and high efficiency of this technique make it a viable diagnostic instrument for a wide range of diseases, thereby fostering the advancement of liquid biopsy [233]. The detection findings won’t be clinically significant unless obtained from body fluids. However, since most body fluids include complex constituents, accurately testing exosomal biomarkers is a considerable difficulty. Enhancing the biosensor’s performance and precision in biological fluids for cancer diagnostics is also necessary [21].

By employing novel signal amplification and detection strategies, ultrasensitive nanosensors enable the detection of zM concentrations. Such sensing capabilities can be extraordinarily beneficial for detecting biomarkers and diagnosing diseases in their nascent stages or post-treatment recurrence. One existing constraint of optical sensing methods when applied in vivo is the significant background fluorescence emitted by tissues within this particular wavelength range. One possible resolution to this issue involves the utilization of upconverting NPs. Although they can be excited in the low energy range, these particles emit a visible-range signal after absorbing multiple photons. One benefit is that excitation occurs via photons in the NIR, a region where tissues are transparent, while detection occurs via visible light. This permits both sensitive detection in the visible spectrum and profound tissue penetration. It is of utmost importance that the upconversion for these particles is efficacious. For this to occur, the material must possess low lattice phonon energies, high chemical stability, and low lattice symmetry. Materials highly compatible with the requirements are tiny salt crystals doped with rare earth atoms, such as Er^3+^, Tm^3+^, and Ho^3+^ ions (often fluorides, chlorides, or bromides). For efficient inclusion, the anion should generally possess a dimension that is comparable to the rare earth ions that are being incorporated. An additional benefit of these UCNPs is their compatibility with contrast agents utilized in alternative imaging modalities (e.g., MRI contrast agents), enabling them to be observed using various techniques. Presently, the most significant obstacle for UCNP is their intricate composition, which makes them prohibitively expensive or challenging to obtain. Despite the positive evaluation of safety and toxicity thus far, there remains an absence of data, particularly about in vivo testing. It has also been determined that controlling size and shape to achieve reproducibility is a significant challenge [234].

## Future and landscape

Over the past decade, interest in the diagnostic applications of EXOs has increased significantly. Numerous research organizations and laboratories are devoted to the enhancement of EXO-based diagnostics. This is because, relative to healthy cells, malignancies secrete an increased quantity of EXOs. In addition to manifesting tumor-specific surface markers, these EXOs transport tumor-specific RNA, DNA, and proteins throughout the body. With such advantageous characteristics, it is unsurprising that EXOs have emerged as a focal point in the ongoing effort to improve tumor diagnostics. This is evident from the fact that all 37 clinical trials listed on the NIH clinical trials website are evaluating the diagnostic efficacy of EXOs derived from tumors. Notwithstanding the constraints inherent in existing techniques for EXO extraction and analysis, several ongoing clinical trials are assessing the effectiveness of EXOs in tracking patients’ progression through cancer treatments. These trials employ a range of nanotechnology-based and chip-based approaches in addition to conventional EXO profiling methods [20].

Optical nanobiosensors may be a viable substitute to surmount these constraints. A broad family of sensors, optical nanobiosensors may recognize various analytes by luminescence, dispersion, refraction, and absorption. Most optical biosensors are constructed using biomarkers, such as microRNAs, EXOs, cancer antigens, circulating tumor cells (CTCs), and proteins. These materials may be taken from body fluids such as blood, saliva, urine, and serum [235]. Consequently, optical biosensors employ a noninvasive methodology. Additional advantages of optical nanobiosensors consist of their exceptional selectivity, minimal sample volume, LOD, dependable nature, economical nature, expeditious detection, and real-time monitoring of the target biomolecules [179]. A straightforward and easy-to-use sensing device, the small SPR biosensor provides sensitive, label-free, real-time, and affordable exosomal protein biomarker detection. It might function as a liquid biopsy assay to support more intrusive and costly diagnostic procedures and help with prognostication, therapy response monitoring, and cancer screening [230].

In contrast to traditional approaches, nanobiosensor technologies offer tremendous promise, including label-free detection, high-throughput screening, real-time analysis, and the requirement for a minute sample quantity for analysis. Extensive research has been conducted on the utilization of nanosized materials (e.g., 1–100 nm) sourced from either organic or inorganic materials to modify electrode surfaces. The objective of this research is to develop biosensors that exhibit enhanced reproducibility, selectivity, and sensitivity [107, 236]. Fast and early diagnosis at the optimal time for therapeutic intervention is closely linked to the best results for specific disease conditions. The development of label-free detection techniques sensitive to minute amounts of analytes found in the physiological environment has been the main focus of recent advances in clinical diagnostics. Real-time, label-free, sensitive, and selective target molecule detection is made possible by optically based detection systems, which are potent instruments. As a result, smaller concentrations of the target molecules may be detected. This results in the use of these sensors as a biomarker-related diagnostic tool. In addition, they are inexpensive, portable, sensitive gadgets with the potential to transform the medical sector completely. Biomaterials are expensive; however, the cost is significantly lowered by the developed sensors’ repeatability. Biomarker analysis is made possible by high-sensitivity optical-based sensors with low detection limits [237]. On numerous occasions, NPs have demonstrated their capacity to surmount certain challenges associated with liquid biopsy analysis. In biosensing, their surface-to-volume ratio, multiplexing capability, and stable optical properties are significant advantages. Nevertheless, despite the intricate nature of the majority of biological fluids, attaining precise and high-sensitivity biomarker targeting remains challenging due to many factors, including but not limited to sample autofluorescence and immunoassay crosstalk. The majority of current research focuses on creating new detection mechanisms and enhancing the LOD of those that already exist. Shortly, a shift towards more point-of-care-oriented sensors is anticipated, making cancer diagnostics simpler, less time-consuming, and possibly more affordable for the general public. Future optical nanobiosensors may benefit from the development of in vivo fluorescent imaging of cancer EXOs, but more research on the cytotoxicity of the NPs will be necessary for this. For the time being, the emphasis will be on facilitating the synthesis and functionalization of those particles and boosting their fluorescence via the combination of other enhancing processes or an increase in quantum yield [144, 238].

Furthermore, to be utilized in blood serum, which represents intricate conditions, the detection system must be meticulously designed, with biorecognition activity being enhanced. Researchers are convinced that research into the utilization of nanomaterial-based optical biosensors for detecting exosomal cancer biomarkers will ultimately develop into a practical and efficient analytical instrument capable of addressing numerous challenges in the future, thereby advancing human health [239].

Even though EXO detection has advanced significantly, there are still some issues that need to be resolved. For instance, suitable modification with biocompatibility polymers or other materials should increase the durability of nanomaterials and selectivity for complicated samples. Digesting enzymes in actual samples may cause problems for biorecognition components used in bioassays. The main barrier to translating laboratory research into clinical practice is the need to establish uniform technical requirements, which include gathering biological samples, separating EXOs from liposomes, and performing detection procedures. Improving the findings’ comparability and creating a trustworthy, extensive dataset will be helpful for EXO research in the future. EXO separation and detection in a high-throughput, cost-effective manner is helpful for simultaneously identifying many clinical samples. With the combined and relentless efforts of several sectors, including chemistry, materials science, and clinical diagnostics, Experimenters think EXO detection using nanomaterials would advance further [22].

## Conclusions

Our main focus in this extensive study is on detecting cancer EXOs using optical nano biosensors. We aim to provide insightful information on ongoing initiatives to solve basic issues and investigate novel approaches for several NP-based optical biosensors for cancer diagnosis. A comprehensive overview of the progressions made in this domain holds importance in the advancement of novel cancer diagnostic instruments. Tumor-derived EXOs are implicated in various aspects of cancer progression, including metastasis, angiogenesis, immune suppression, and aberrant cell proliferation. Its molecular cargo contains an abundance of biomarkers for malignancy. Several sophisticated molecular profiling techniques aid in comprehensively investigating EXOs derived from tumors. This establishment establishes a solid groundwork for the detection and identification of biomarkers that are more precise and effective. The increasing recognition of the significance of tumor-derived EXOs in the scientific community is due to their potential application in cancer therapy; numerous clinical trials have been initiated utilizing these EXOs as evidence. The advancement of techniques for isolating, purifying, and modifying EXOs provides a chance to maximize their use in a therapeutic setting. EXO-based detection techniques are valuable, but because of their tiny size, they have significant false-positive rates, need considerable sample purification, and provide labeling challenges. Furthermore, by integrating with several complex DNA- or enzyme-based signal amplification techniques, the sensitivity and selectivity of biosensors for EXO detection have significantly increased. Considering the optical techniques based on light scattering, microvesicles’ relative and absolute size distributions may be measured in a matter of minutes by DLS and NTA, respectively. Without labeling, single microvesicles’ size, concentration, and biochemical makeup may be determined using Raman spectroscopy; however, the measuring process takes hours. In optical assays, nanomaterials possessing remarkable luminescence characteristics have emerged as a significant substitute for conventional dyes due to their adjustable emission wavelength, high quantum yield of luminescence, and favorable photostability. QDs, including carbon and silicon QDs, have been applied to EXO imaging and have demonstrated promising potential for EXO sensing. The unique optical properties of noble metal NPs, which result from their interactions with incident light, have found extensive application in various fields. Notably, Ag and Au NPs have been extensively utilized to augment the signal intensity in SPR and SERS assays. Several (combinations of) techniques can, in conclusion, accurately identify clinically significant characteristics of EXOs and microparticles. These methods ought to be investigated further and validated through the comparison of measurement outcomes to achieve analyses that are precise, dependable, and rapid. Optical nanobiosensors are a promising new tool for detecting extracellular cancer biomarkers such as EVs, circulating tumor DNA, and cancer-associated proteins because of their remarkable sensitivity, specificity, and multi-molecule recognition abilities. Researchers could distinguish between CDE using our optical nanosensor by directly detecting several biomarkers, including miRNA, RNA, DNA, proteins, and antibodies inside the EXOs, as well as by identifying certain exosomal membrane proteins. The creation of quick and effective exosomal biomarkers originating from cancer as well as EXO biosensing nanoplatforms are made possible by these nanosensor designs. Future developments in optical nanosensors, contrast agents, molecular techniques, artificial intelligence, and cancer biomarkers using EXOs will facilitate the real-time detection of signals unique to cancer. Risk-based diagnosis and prevention must be widely available and reasonably priced to lessen the toll that cancer takes on society. It is noteworthy that the topic of developing nanosensors is very multidisciplinary, including knowledge from many fields like materials science, biology, chemistry, physics, nanotechnology, and engineering. By resolving these issues, scientists and technologists may further enhance optical nanosensors, augmenting their potential and permitting a wider implementation in the identification of cancer EXOs. Optical nanobiosensor-based cancer EXO detection has to overcome many translational barriers before it can become a useful therapeutic tool. These include standardizing methods, validating across large patient groups, and integrating seamlessly with existing diagnostic processes. To close the gap between laboratory-based research and clinical application, researchers and clinicians must work closely together. Through the combined and relentless efforts of several sectors, including chemistry, materials science, and clinical diagnostics, we think that EXO detection using nanomaterials will advance further.

## Data Availability

No datasets were generated or analysed during the current study.
